# Tracking pairwise genomic loci by the ParB–ParS and Noc-NBS systems in living cells

**DOI:** 10.1093/nar/gkae134

**Published:** 2024-02-27

**Authors:** Xiaohui He, Yuxi Tan, Ying Feng, Yadong Sun, Hanhui Ma

**Affiliations:** Gene Editing Center, School of Life Science and Technology, ShanghaiTech University, Shanghai, China; Gene Editing Center, School of Life Science and Technology, ShanghaiTech University, Shanghai, China; Gene Editing Center, School of Life Science and Technology, ShanghaiTech University, Shanghai, China; School of Life Science and Technology, ShanghaiTech University, Shanghai, China; Gene Editing Center, School of Life Science and Technology, ShanghaiTech University, Shanghai, China

## Abstract

The dynamics of genomic loci pairs and their interactions are essential for transcriptional regulation and genome organization. However, a robust method for tracking pairwise genomic loci in living cells is lacking. Here we developed a multicolor DNA labeling system, mParSpot (multicolor ParSpot), to track pairs of genomic loci and their interactions in living cells. The mParSpot system is derived from the ParB/ParS in the parABS system and Noc/NBS in its paralogous nucleoid occlusion system. The insertion of 16 base-pair palindromic ParSs or NBSs into the genomic locus allows the cognate binding protein ParB or Noc to spread kilobases of DNA around ParSs or NBSs for loci-specific visualization. We tracked two loci with a genomic distance of 53 kilobases and measured their spatial distance over time. Using the mParSpot system, we labeled the promoter and terminator of the *MSI2* gene span 423 kb and measured their spatial distance. We also tracked the promoter and terminator dynamics of the *MUC4* gene in living cells. In sum, the mParSpot is a robust and sensitive DNA labeling system for tracking genomic interactions in space and time under physiological or pathological contexts.

## Graphical Abstract

**Graphical Abstract:**
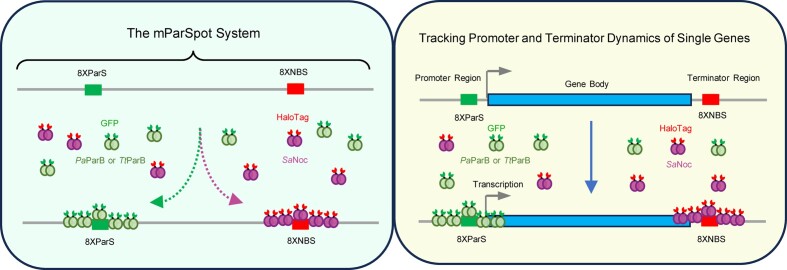

## Introduction

Chromatin carries genetic information and is hierarchically folded in the nucleus (1). The spatiotemporal organization of chromatin is essential for transcriptional regulation during the cell cycle progression, stem cell differentiation, and animal development (2,3). It has been shown that the dynamic interaction between enhancer and promoter regulates the transcriptional activity (4). Labeling pairs of regulatory elements of genes in living cells is critical for exploring the role of genomic interactions in transcriptional regulation and cellular functions. RNA polymerase II (RNAPII) is a multiunit protein complex for the transcription of mRNA, snRNA, and microRNA precursors (5). How RNAPII initiates and executes the RNA transcription of the active genes in living cells is largely unknown.

The fluorescence repressor operator system (FROS) was developed to visualize DNA by integration of a 256-lac operator repeat into CHO cells or yeast, and recruiting its binding partner lac repressor fused with GFP (6). Nevertheless, the 256-LacO repeats reach 10 kb in length (6), which is not trivial to integrate into the human or mouse genome (7). The integration of a large number of LacO repeats has been shown to affect chromatin structure and gene expression (8). Later, the TetO array and CuO array were utilized to study the enhancer and promoter interaction of the Sox2 gene (9). TetO and its variant mut-TetO have been used to track genomic interactions during VDJ recombination in live cells (10). The requirement of LacO repeat numbers for visualization has decreased to as low as 20 copies in Drosophila, but the signal-to-noise ratio is relatively low (11). To achieve a high signal-to-noise ratio, the requirement of large-size DNA repeat integration for FROS hampers its widespread applications of genomic DNA imaging.

CRISPR-based DNA imaging was developed by the fusion of GFP to nuclease dead Cas9 (dCas9) along with the single guide RNA (sgRNA) targeting the tandem repeats in the human genome (12). With the optimization of the sgRNA scaffold, multiple repetitive genomic loci were simultaneously labeled in live cells by CRISPRainbow or CRISPR-Sirius (13,14). For imaging of non-repetitive DNA by dCas9-GFP, 26 sgRNAs initially or 12 sgRNAs later were required to visualize a genomic locus (12,15). Further elaborating the dCas9-based DNA imaging systems have allowed one or two sgRNAs to image non-repetitive genomic locus, primarily by liquid-liquid phase separation (LLPS)-mediated signal amplification (16–18). However, it is hard to control the locus-specificity and the degree of signal amplification by phase separation. The R-loop formed by dCas9 and sgRNA targeting may also result in transcriptional repression or genome instability (19). TriTag (20), a dCas9-based DNA imaging tool, allows the visualization of DNA, RNA and protein simultaneously. The strategy of DNA visualization by TriTag is similar to FROS, which is required to integrate DNA elements for recognition but without signal amplification.

An alternative method for live-cell DNA imaging is the ParB/ParS-based ANCHOR system, which enables the labeling of DNA efficiently with minimal disruption of transcription or genomic stability (21,22). The insertion of 16 base-pair palindromic ParSs into a genomic locus allows the ParB protein to spread kilobases of DNA around ParS for loci-specific visualization (23). This approach has been applied to visualize gene transcription or viral replication in mammalian cells (21,24). Recently the ANCHOR system has been used to study 3D genome organization in drosophila or mammalian cells (25–27). ANCHOR3 has been optimized to use in mammalian cells, and ANCHOR1 has recently been used in mESCs with a low signal-to-noise ratio (28). Thus, it is important to develop a multicolor ParB/ParS system for live-tracking multiple or pairs of genomic loci.

Here we developed a multicolor ParSpot system based on the ParB/ParS and Noc/NBS systems for tracking genomic interactions in living cells. The ParSpot system is derived from the parABS system (ParB–ParS) and its paralogous nucleoid occlusion system (Noc-NBS). We found the ParB–ParS system from *P. aeruginosa* or *T. thermophilus* and the Noc-NBS system from *S. aureus* could simultaneously label pairs of genomic loci with high efficiency. We tracked pairs of loci with various genomic distances and measured their spatial distance over time or their colocalization with Pol II clusters. Thus, we believe the multicolor ParSpot is a robust and sensitive DNA labeling system for exploring 3D genome organization in living cells.

## Materials and methods

### Plasmid construction

Human Codon-optimized *Bc*ParB1, *Bc*ParB2, *Bs*ParB, *Hh*ParB, *Pa*ParB, *Tt*ParB, *Bs*Noc, *Cd*Noc, *Gt*Noc, *La*Noc, *Sa*Noc, and TdStaygold DNA fragments were synthesized by GENEWIZ, Inc. To construct ParB or Noc-HaloTag expression plasmids, orthogonal ParB or Noc was cloned into pHAGE-EFS-MCP-HaloTag by NcoI and BamHI, resulting in pHAGE-ParB-HaloTag or pHAGE-Noc-HaloTag. Donor plasmids for 8XParS or 8XNBS integration include 8XParS or 8XNBS, puromycin or hygromycin expression cassette, and homolog recombination arms for the P1, P2, P3, P4, P5 or P6 sites. The 8X*Bc*ParS1, 8X*Bc*ParS2, 8X*Bs*ParS, 8X*Pa*ParS, 8X*Tt*ParS, 8XParSc and 8XNBSc (the sequences shown in Supplementary Table S1) were synthesized by Beijing Tsingke Biotech Co., Ltd. The puromycin expression cassette includes the CMV promoter, puromycin coding sequence and SV40 polyA signal (P_CMV_-Puro-PolyA_SV40_). The hygromycin expression cassette includes the EFS promoter, hygromycin coding sequence and SV40 polyA signal (P_EFS_-Puro-PolyA_SV40_). The left arm (Larm) and right arm (Rarm) for homolog recombination with the P1, P2, P3, P4, P5 or P6 sites were amplified from U2OS genomic DNA prepared by a cell DNA isolation mini kit (Vazyme Biotech Co.). The DNA fragments including Larm- P1, P2, P3, P4, P5 or P6, 8XParS or 8XNBS, P_CMV_-Puro-PolyA_SV40_, Rarm P1, P2, P3, P4, P5 or P6 were generated by 2X MultiF Seamless Assembly (Abclonal Inc.), which were cloned into pDONOR3.1 by MluI and EcoRI, resulting in pDONOR-Larm-8XParS or 8XNBS-P_CMV_-Puro-PolyA_SV40_-Rarm- P1, P2, P3, P4, P5 or P6. To generate the HaloTag donor plasmids for integration into the N-terminal of pol II subunit RPB1, HaloTag along with the left and right arms were cloned into pDONOR3.1 by MluI and EcoRI, resulting in pDONOR-Larm-HaloTag-Rarm-RPB1. To generate sgRNA expression plasmids for genomic integration, the guide RNA targeting P1, P2, P3, P4, P5 or P6 or RPB1 was subcloned into pLH-sgRNA1 by Bbs I, resulting in pLH-sgRNA1- P1, P2, P3, P4, P5 or P6 or RPB1. The guide RNA targeting C3 for imaging was subcloned into pLH-sgRNA2 by Bbs I, resulting in pLH-sgRNA2-C3. The SgRNA sequence is in Supplementary Table S2.

### Generation of U2OS-8XParS or 8XNBS cell lines

U2OS cells were cultured on 10 cm dishes at 37°C in DMEM with high glucose (Life Technologies) supplemented with 10% (vol/vol) FBS. To generate U2OS-8XParS or 8XNBS cell lines, U2OS cells were co-transfected with 1 μg of pHAGE-Cas9-P2A-sfGFP, 600 ng of pLH-sgRNA1- P1, P2, P3, P4, P5 or P6 and 500 ng of pDONOR-8XParS or 8XNBS- P1, P2, P3, P4, P5 or P6 using Lipofectamine 2000 (Life Technologies) for 6 h and then replaced with fresh culture media. The transfected cells were cultured for an additional 48 hours before flow cytometry to sort BFP and GFP double-positive cells by FACSAria III or FACSAria Fusion Cell Sorter. The collected cells were plated on 48-well plates and cultured for an additional 24 h. Puromycin or Hygromycin was added to enrich the cells with 8XParS or 8XNBS integration for 7 days. Single cells were then sorted into 96-well plates and cultured for an additional two to three weeks. the single-cell clones were expanded and subjected to genotyping. Clonal genotyping was performed using the primers in Supplementary Table S3.

### Generation of U2OS-RPB1-HaloTag cell lines

To study the colocalization of promoters and Pol II clusters, HaloTag was integrated into the N-terminal of Pol II subunit RPB1. U2OS cells were co-transfected with 1 μg of pHAGE-Cas9-P2A-sfGFP, 600 ng of pLH-sgRNA1-RPB1 and 500 ng of pDONOR-Larm-HaloTag-Rarm-RPB1 using Lipofectamine 2000 (Life Technologies) for 6 h and then replaced with fresh culture media. The transfected cells were cultured for an additional 48 hours and replaced with media containing 2 nM HaloTag-JF549 16 hours before imaging. Fluorescence imaging was used to check the proper localization of HaloTag-RPB1. Single cells with HaloTag-positive were then sorted into 96-well plates and cultured for an additional 2–3 weeks. The single-cell clones were expanded and subjected to genotyping. Clonal genotyping was performed using primers in Supplementary Table S3.

### Labeling genomic loci by the mParSpot system

To examine whether U2OS-8XParS or 8XNBS cells could be used for genomic loci labeling, ParB- or Noc-HaloTag was transiently expressed in these cells. 500 ng of orthogonal pHAGE-ParB-HaloTag and Noc-HaloTag, 600 ng of pLH-sgRNA2-C3 and 300 ng of pHAGE-dCas9-GFP were co-transfected using Lipofectamine 2000 (Life Technologies) for 6 h and replaced fresh media for an additional 48 hours. The transfected cells were replaced with media containing 2 nM HaloTag-JF549 16 h before imaging. To label loci pairs by the mParSpot system in the U2OS-8XNBSc-8XParSc cells, 500 ng of pHAGE-*Sa*Noc-TdStaygold, 500 ng of pHAGE-*Tt*ParB-HaloTag, 600 ng of pLH-sgRNA2-C3 and 300 ng of pHAGE-dCas9-BFP were co-transfected using Lipofectamine 2000 (Life Technologies) for 6 hours and replaced fresh media for an additional 48 h. The transfected cells were replaced with media containing 2 nM HaloTag-JF549 16 hours before imaging. To track the dynamics of two adjacent promoters by mParSpot, 500 ng of pHAGE-*Sa*Noc-TdStaygold and 500 ng of pHAGE-*Pa*ParB-SNAP were co-transfected into U2OS-8XNBSc-8XPaParS-RPB1-HaloTag cells using Lipofectamine 2000 (Life Technologies) for 6 h and replaced fresh media for an additional 48 h. The transfected cells were replaced with media containing 2 nM HaloTag-JF549 and 5 nM SNAP-Cell® 647-SiR 16 h before imaging.

### RT-qPCR

The cells were seeded onto 6 cm culture dishes the day before transfection. 1.3 μg of LacI, *Tt*ParB or *Sa*Noc plasmids are transfected into 8XLacO, 8XParSc or 8XNBSc cells respectively. TdMCP plasmid was as the control group. Cells were collected to sort positive cells by FACS. We use the RNAprep Pure Micro Kit (Tiangen Biotech (Beijing) Co.,Ltd.) to extract total RNA. The RT-qPCR reaction was performed using the kit of ABScript III RT Master Mix for qPCR with gDNA Remover and 2X Universal SYBR Green Fast qPCR Mix (ABclonal). The experimental primers for PPP1R2 are 5′-GAAGA TGCCTGTAGT GACACCG-3′ and 5′-CGTTCTTCAGGTGAGAGGTCAC-3′. The control primers for GAPDH are 5′-CAATGACCCCTTCATTGACC-3′ and 5′-TTGATTTTGGAGG GATCTCG-3′.

### Fluorescence microscopy

The live cell imaging was carried out on a DeltaVision Ultra imaging system (Leica) equipped with a 100X oil objective lens (NA 1.4), equal to a pixel size of 64.5 nm in the image. The cells were cultured on No. 1.0 glass bottom dishes (MatTek). The microscope stage incubation chamber was maintained at 37°C and 5% CO_2_. BFP was excited with an excitation filter at 397/31 nm, and its emission was collected using an emission filter at 438/36 nm. sfGFP or TdStaygold was excited at 478/28 nm and collected using the filter at 512/23 nm. HaloTag-JF549 was excited at 548/34 nm, and its emission was collected using the filter at 592/38 nm. SNAP-Cell® 647-SiR was excited with an excitation filter at 633/27 nm, and its emission was collected using an emission filter at 677/46 nm. The fluorescence Imaging data were acquired by DeltaVision Elite imaging (Leica) software. The images were captured in z-stacks with an exposure time of 50 ms under 50% laser power for HaloTag, GFP, BFP or SNAP respectively. The step size in z-stacks was 200 nm. To detect locus numbers, maximum intensity projection of z-series images was performed. Image size was adjusted to show individual nuclei, and intensity thresholds were set based on the ratios of P1 focal signals to nucleoplasm fluorescence. For the live tracking, images from different colors were acquired for 100 s in Supplementary video S1, 50 s in Supplementary video S2, and 80 s in Supplementary video S3. The videos were generated by using ImageJ software and the video play rate is 15 fps (frame per second). For the representative images, the raw data were deconvoluted by softWoRx (Leica) software. To see the merged images among 8XParS, 8XNBS or C3 foci more clearly, we readjusted the contrast in the zoom images.

### Labeling efficiency and signal-to-noise (SNR)

The P1 foci were defined by their colocalization with the C3 repeat, i.e. the spatial distance between P1 and C3 is less than 500 nm. The labeling efficiency was estimated by the percentage of cells shown the P1 foci. Signal-to-noise (SNR) of genomic loci labeling was calculated as the ratio of fluorescence intensity from the signal (P1 locus) and nucleoplasmic background. The SNR was calculated with the formula: SNR = (*I*_S_ − I_B_)/(*I*_N_− I_B_). *I*_S_ is the intensity of the labeled P1 loci; *I*_N_ is the intensity of the nucleoplasm; and *I*_B_ is the background fluorescence intensity from a dark region in the same image.

### Spatial distance analysis

To quantify the spatial distance or track the dynamics, we analyzed pairs of loci lying in the same focal plane. The raw data was deconvoluted and projected by softWoRx (Leica) software. To measure the spatial distance of P1 and P2 locus in Figure 5, P3 and P4 locus in Figure 6, images were processed by ImageJ/Fiji. Distances were calculated with the formula: $D = \sqrt[2]{{{{( {X2 - X1} )}}^2 + {{( {Y2 - Y1} )}}^2}}$.

### 3D distance calculation

The 3D distance in Figure 7D was analyzed by Imaris. We use the spot module to mimic and locate the P5 and P6 loci. After location, we use the plug-in of shortest distance to spots to record the 3D distance between P5 and P6.

### Statistical analysis

All box plots and bar graphs were generated using GraphPad Prism. The exact n values used to calculate statistics are described in the associated figure legends. Error bars represent as standard deviation (SD) from data in at least triplicate experiments. All the images and videos shown in the figures were repeated at least three times independently with similar results.

## Results

### Live-cell DNA imaging by orthogonal ParB–ParS systems

The ParB–ParS system has been repurposed for DNA imaging in living cells, termed the ANCHOR system (21,22). To study the genomic interactions, labeling pairs of loci by orthogonal ParB–ParS systems is required. The ParB–ParS system is derived from ParABS, a conserved system for chromosome segregation and plasmid partitioning in bacteria (29). Here we chose five orthogonal ParBs and their corresponding ParSs including *Bc*ParB1/*Bc*ParS1, *Bc*ParB2/*Bc*ParS2 from *B. cenocepacia* (23), *Bs*ParB/*Bs*ParS from *B. subtilis* (30), *Pa*ParB/*Pa*ParS from *P. aeruginos* (31), *Tt*ParB/*Tt*ParS from *T. thermophilus* (32), and *Hh*ParB from *H. hemicellulosilytic* with consensus ParS (ParSc).

**Figure 1.**
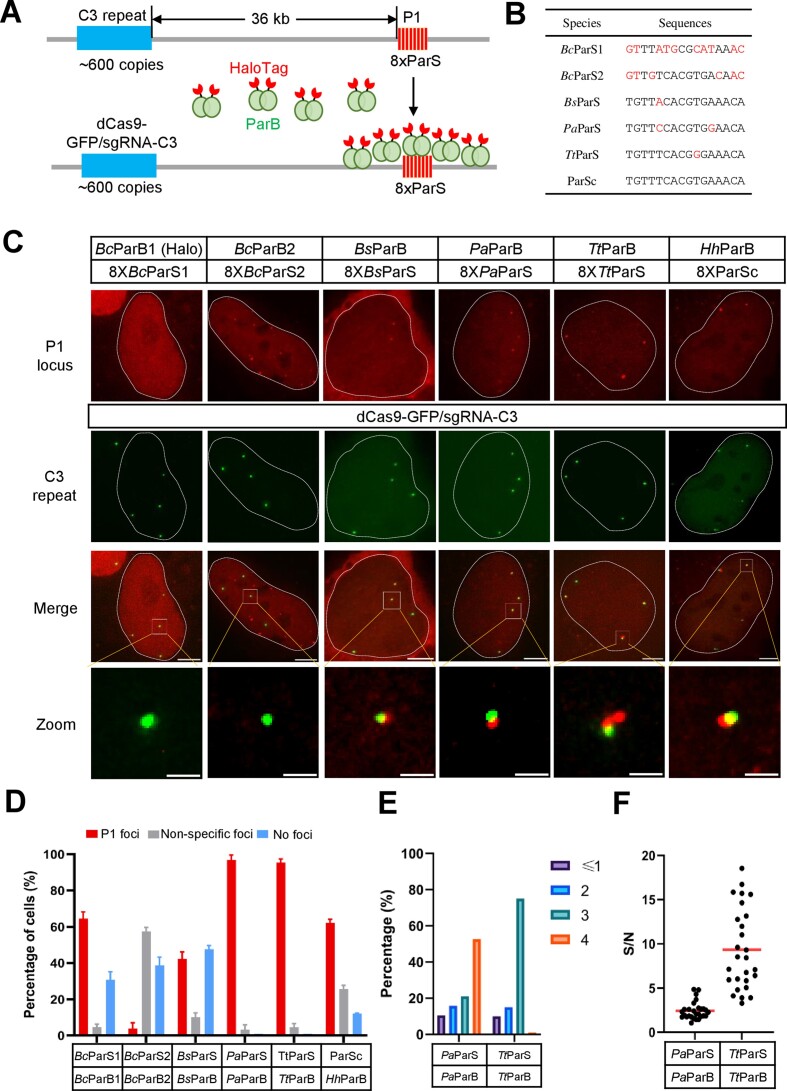
Imaging site-specific genomic DNA by orthogonal ParB–ParS systems. (**A**) Schematic of DNA labeling by the ParB–ParS system. 8XParS (8 copies) was inserted into 36 kilobases downstream of the C3 repeat (600 copies) in U2OS cells. The genomic locus inserted with 8XParS was termed P1. ParB-HaloTag was used to label the P1 locus and dCas9-GFP/sgRNA-C3 was used to visualize C3 repeat. (**B**) ParS sequences from different bacterial species. *Bc*ParB1 and *Bc*ParS2 are from *B. cenocepacia*, *Bs*ParS from *B. subtilis*, *Pa*ParS from *P. aeruginosa* and *Tt*ParS from *T. thermophilus*, ParSc is the ParS consensus sequence. (**C**) Loci-specific labeling by orthogonal ParB–ParS. 8X*Bc*ParS1, 8X*Bc*ParS2, 8X*Bs*ParS, 8XParSc, 8X*Pa*ParS or 8X*Tt*ParS was integrated into 36 kilobases downstream of the C3 repeat in U2OS cells. The integration site (P1 locus, red) was visualized by their cognate ParB-HaloTag along with CRISPR-based labeling of C3 repeat (green). The scale bars are 5 μm for the whole cell and 1 μm for the zoom images. (**D**) The percentage of specific labeling cells by orthogonal ParB–ParS. The percentage of cells with specific P1 foci was shown in red. The percentage of cells with no foci was shown in blue and cells with non-specific foci was shown in grey. n = 36 cells for *Bc*ParS1/*Bc*ParB1, 16 for *Bc*ParS2/*Bc*ParB2, 42 for *Bs*ParS/*Bs*ParB, 20 for *Pa*ParS/*Pa*ParB, 29 for *Tt*ParS/*Tt*ParB, 25 for ParSc/*Hh*ParB. (**E**) The foci numbers of orthogonal ParB–ParS from single cell clones. *n* = 20 cells for each group. (**F**) The signal-to-noise ratio of labeling by orthogonal ParB–ParS. The signal-to-noise (S/N) ratio of each ParB–ParS pair was measured. Each data point represents the S/N of each P1 labeling. The red bar represents the average of S/N in U2OS cells. *n* = 28 for *Pa*ParS/*Pa*ParB group, 27 for *Tt*ParS/*Tt*ParB group.

To examine whether these ParB/ParS systems can be repurposed for DNA imaging, we inserted octets of ParS palindrome sequence (8XParS) 36 kilobases downstream a repetitive region (C3 repeat, ∼600 copies) (13) in chromosome 3, and named the P1 locus. C3 repeat is labeled by dCas9-GFP/sgRNA-C3 and 8XParS is labeled by HaloTag-fused ParB (Figure 1A, Supplementary Figure S1A). As shown in Figure 1B and Supplementary Table S1, the cognate ParS sequence of each ParB consists of 16 nucleotides and the ParS octet consists of eight ParS with a short linker sequence between ParSs. To efficiently integrate 8XParS into the genome of U2OS cells, we added a puromycin expression cassette downstream of 8XParS, and single clones resistant to puromycin were selected (Supplementary Figure S1A, B). Through the puromycin selection, we achieved up to 90% of single colonies containing 8XParS integration (Supplementary Figure S1C).

To characterize the labeling specificity, the colocalization of C3 repeat (dCas9-GFP/sgRNA-C3) and P1 locus (ParB-HaloTag/8XParS) was measured. *Bc*ParB1/*Bc*ParS1 (ANCHOR1) and *Bc*ParB2/*Bc*ParS2 (ANCHOR2) have been successfully used to label DNA in yeast or drosophila, but haven’t been carefully examined in mammalian cells. As shown in Figure 1C, D and Supplementary Figure S2, *Bc*ParB2 and *Hh*ParB show many non-specific foci regardless of the presence or absence of 8XParS in mammalian cells. Specific loci adjacent to C3 repeat were detected when *Bc*ParB1-, *Bs*ParB-, *Pa*ParB-, *Tt*ParB- or *Hh*ParB-HaloTag was transfected into U2OS cells integrated with 8X*Bc*ParS1, 8X*Bs*ParS, 8X*Pa*ParS, 8X*Tt*ParS or 8XParSc respectively. The statistical data in Figure 1D showed that the percentage of cells with specific P1 labeling is high with *Pa*ParB/8X*Pa*ParS (96.8%) or *Tt*ParB/8X*Tt*ParS (95.4%), while a high percentage of cells with no foci labeling at P1 when using *Bc*ParB1/8x*Bc*ParS1 (30.8%) or *Bs*ParB/8x*Bs*ParS (47.6%). Thus, we chose *Pa*ParB/8X*Pa*ParS and *Tt*ParB/8X*Tt*ParS for the following studies.

To examine the homogeneity of labeling efficiency from single colonies, we counted the P1 foci number in *Pa*ParB-HaloTag transfected U2OS-8X*Pa*ParS cell lines or *Tt*ParB-HaloTag transfected U2OS-8X*Tt*ParS cell lines. As shown in Figure 1E, 52.6% of U2OS-8X*Pa*ParS cells contain 4 foci colocalized with all 4 C3 foci, and 75% of U2OS-8X*Tt*ParS cells contain 3 foci colocalized with 3 out of 4 C3 foci. The signal-to-noise (S/N) ratio is 2.4 on average for *Pa*ParB/8X*Pa*ParS and 9.3 on average for *Tt*ParB/8X*Tt*ParS (Figure 1F).

### The low specificity of ParS recognition by Orthogonal ParBs

**Figure 2.**
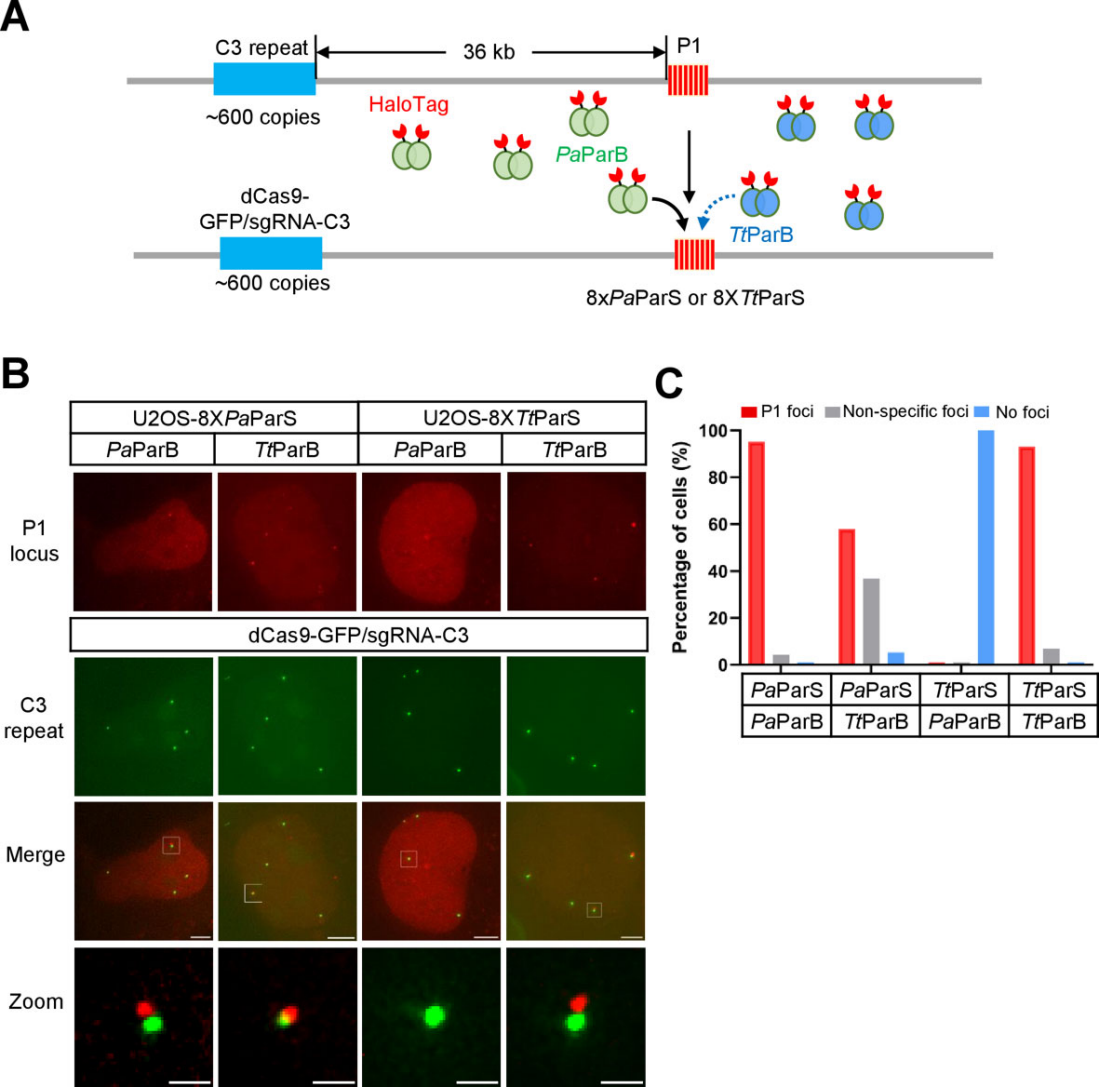
The specificity of ParS recognition by orthogonal ParBs. (**A**) Schematic of ParS labeling by the orthogonal ParBs. 8X*Pa*ParS or 8X*Tt*ParS was inserted into 36 kb downstream of the C3 repeat (600 copies) in U2OS cells. P1 locus was labeled by ParB-HaloTag and C3 repeat was visualized by dCas9-GFP/sgRNA-C3. (**B**) Labeling specificity of 8X*Pa*ParS and 8X*Tt*ParS by *Pa*ParB and *Tt*ParB. The 8X*Pa*ParS or 8X*Tt*ParS integration site (P1 locus, red) was visualized by orthogonal ParB-HaloTag along with CRISPR-based labeling of C3 repeat (green). The scale bars are 5 μm for the cells and 1 μm for the zoom images. (**C**) The percentage of specific labeling cells by orthogonal ParSs and ParBs. The percentage of cells with specific P1 foci was shown in red, non-specific foci in grey, and no foci in blue. *n* = 20 cells in each group.

To examine whether *Pa*ParB/*Pa*ParS and *Tt*ParB/*Tt*ParS could be used for multicolor DNA imaging, we transfected *Tt*ParB-HaloTag into U2OS-8X*Pa*ParS cells or *Pa*ParB-HaloTag into U2OS-8X*Tt*ParS cells (Figure 2A). As shown in Figure 2B and C, the *Tt*ParB-HaloTag was efficiently label the P1 locus (8X*Pa*ParS or 8X*Tt*ParS) in both U2OS-8X*Pa*ParS cells and U2OS-8X*Tt*ParS cells suggesting that *Tt*ParB is lacking the specificity to recognize *Pa*ParS or *Tt*ParS. We also transfected *Bs*ParB-, *Hh*ParB-, *Pa*ParB- or *Tt*ParB-HaloTag into U2OS-8XParSc cells, along with dCas9-GFP/sgRNA-C3 for labeling C3 repeat (Supplementary Figure S3A). The P1 locus (8XParSc) was effectively labeled in U2OS-8XParSc cells when transfected by *Bs*ParB (87.0%), *Hh*ParB (62.2%), *Pa*ParB (85.2%) or *Tt*ParB (93.1%) (Supplementary Figure S3B and S3C, Supplementary Video S1). The P1 foci numbers are different in *Bs*ParB-, *Hh*ParB-, *Pa*ParB- or *Tt*ParB-HaloTag transfected U2OS-8XParSc cells. As shown in Supplementary Figure S3D, 91.3% of U2OS-8XParSc cells contain 4 foci of P1 colocalized with C3 when transfected with *Tt*ParB-HaloTag. Nevertheless, less than 40% of U2OS-8XParSc cells were labeled 4 foci of P1 colocalized with C3 when transfected with *Bs*ParB, *Hh*ParB or *Pa*ParB-HaloTag. These results indicate highly efficient labeling of P1 in U2OS-8XParSc cells can be achieved by *Tt*ParB-HaloTag.

### Live-cell DNA imaging by the Noc-NBS, a paralogous ParB–ParS system

The nucleoid occlusion protein (Noc) is a ParB-related protein sharing similar domain structures but plays different roles in the cell division of bacteria (33). The ParABS (ParA/ParB/ParS) system is critical for chromosome segregation and plasmid partitioning in bacteria. The nucleoid occlusion protein (Noc) binds to and spreads around the specific DNA sequences (NBSs), which protects genomic integrity by controlling DNA replication and chromosome segregation in bacteria.

**Figure 3.**
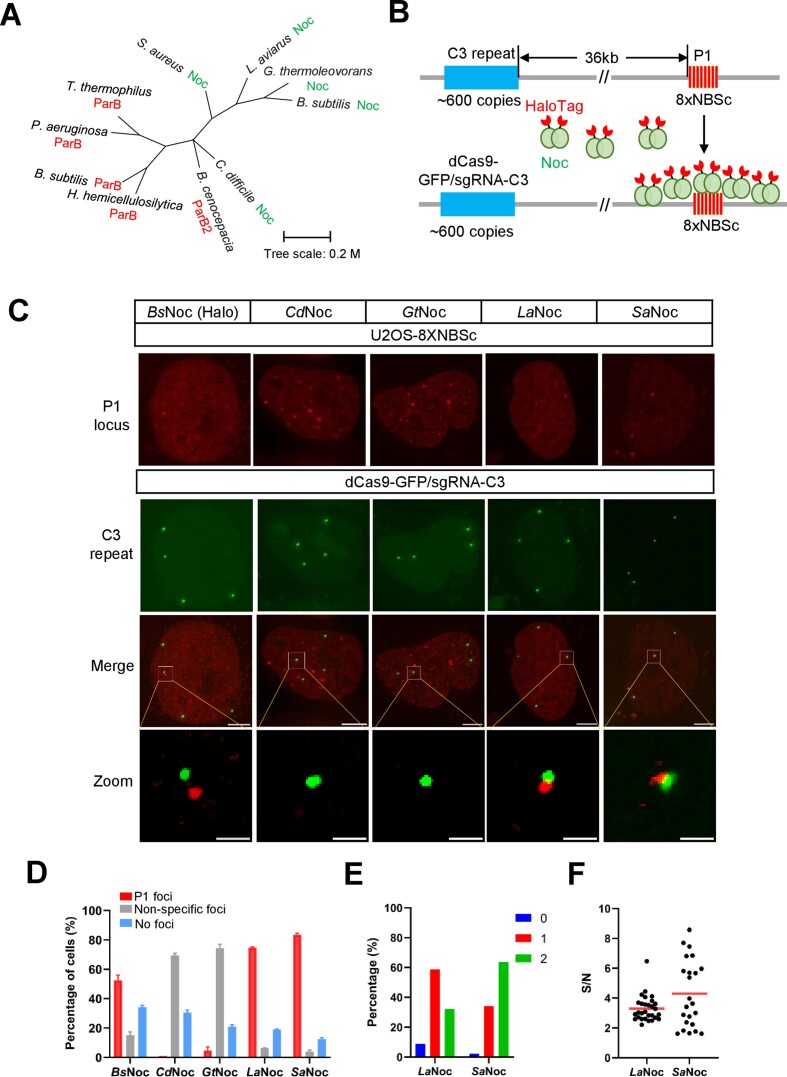
Imaging site-specific genomic DNA by the Noc-NBS system. (**A**) Phylogenetic tree of ParB and paralogous Noc proteins. Tree scale: 0.2 million years. (**B**) Schematic of DNA labeling by the Noc-NBS system. 8XNBSc (8 copies of NBS consensus sequence) was inserted into 36 kb downstream of the C3 repeat in U2OS cells. P1 locus (8XNBSc) was labeled by Noc-HaloTag and C3 repeat was visualized by dCas9-GFP/sgRNA-C3. (**C**) Labeling specificity of NBSc by orthogonal Noc proteins. The 8XNBSc integration site (P1 locus, red) was visualized by orthogonal Noc-HaloTag along with CRISPR-based labeling of C3 repeat (green). The scale bars are 5 μm for the cells and 1 μm for the zoom images. (**D**) The percentage of specific labeling cells by NBSc and orthogonal Noc. The percentage of cells with specific P1 foci was shown in red, no foci in blue, and non-specific foci in grey. *n* = 50 cells in each group. (**E**) The foci numbers of orthogonal Noc-NBS from single cell clones. *n* = 34 for *La*Noc, *n* = 44 cells for *Sa*Noc. (**F**) The signal-to-noise ratio of labeling by NBSc and orthogonal Noc. The signal-to-noise (S/N) ratio of each NBSc and Noc pair was measured. Each data point represents the S/N of each P1 labeling. The red bar represents the average of S/N in U2OS cells. *n* = 29 for *La*Noc, *n* = 23 for *Sa*Noc.

Due to the evolutionary-related ParB and Noc sharing similar mechanisms to bind DNA sequences, we are intrigued about whether ParB–ParS and Noc-NBS could be utilized to generate multicolor DNA imaging systems. Here we selected five Noc proteins from *S. aureus* (*Sa*Noc), *B. subtilis* (*Bs*Noc), *L. aviarus* (*La*Noc), *C. difficile* (*Cd*Noc) and *G. thermoleovorans* (*Gt*Noc) (34,35) for DNA labeling test (Figure 3A). As illustrated in Figure 3B, we integrated octets of an NBS consensus sequence (8XNBSc) 36 kilobases downstream of the C3 repeat, which has been named the P1 locus. The P1 locus with 8XNBSc is labeled by HaloTag-fused Noc. As shown in Figure 3C and Supplementary Figure S4, many non-specific foci were observed in *Cd*Noc or *Gt*Noc transfected U2OS cells regardless of the presence or absence of 8XNBSc. On the contrary, specific loci adjacent to C3 repeat were detected when *Bs*Noc, *La*Noc or *Sa*Noc was transfected into U2OS-8XNBSc cells. The statistical data in Figure 3D showed that the percentage of cells with specific P1 labeling is 52.5% for *Bs*Noc, 74.7% for *La*Noc or 83.6% for *Sa*Noc respectively. We found that *Sa*Noc can label 2 P1 foci in 63.6% of U2OS-8XNBSc cells with a signal-to-noise (S/N) ratio of 4.3 on average (Figure 3E and F). Due to the high specificity and superior signal-to-noise ratio, we chose *Sa*Noc/8XNBSc for the following studies.

### The High specificity of ParB and ParS or Noc and NBS recognition

**Figure 4.**
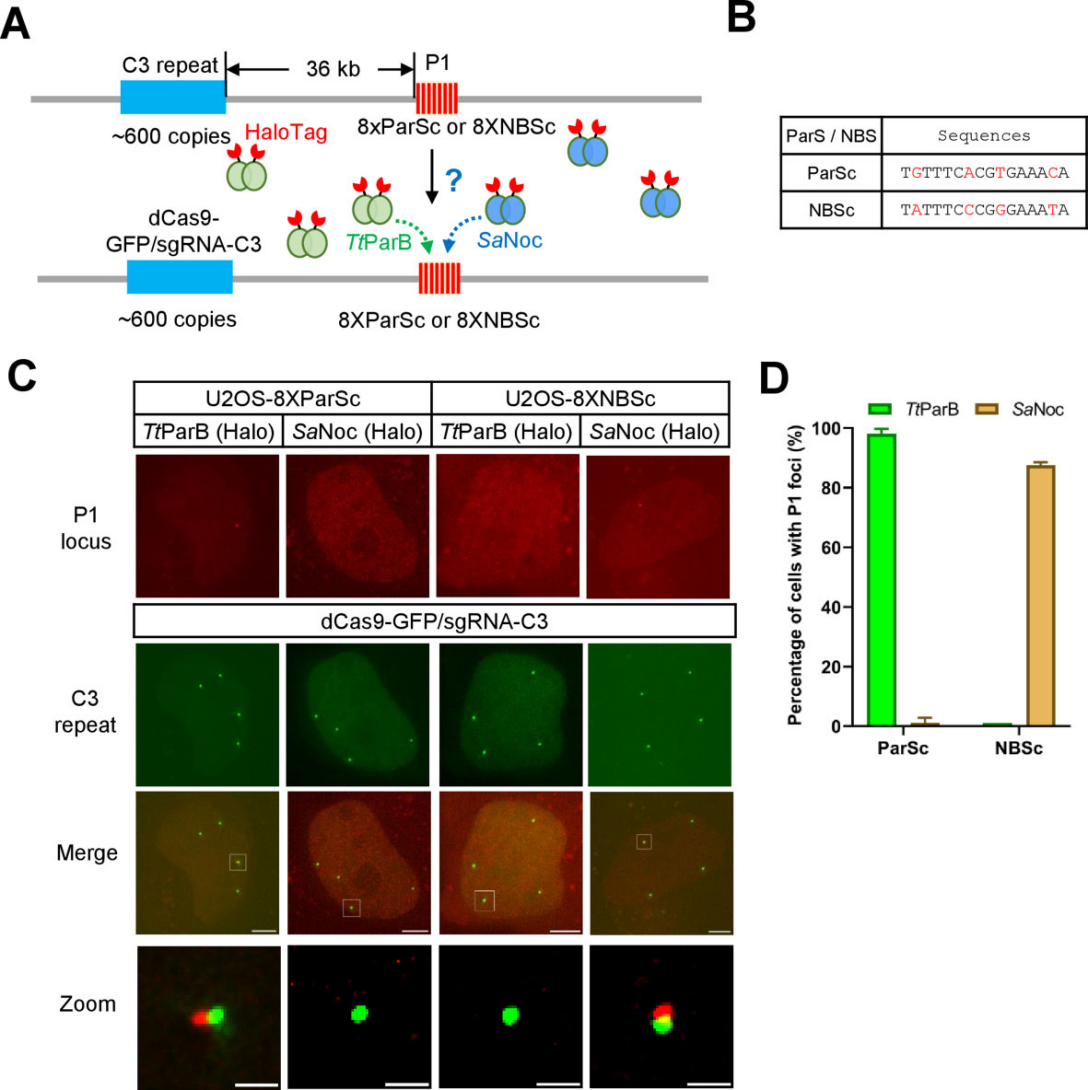
The specificity of ParS or NBS recognition by ParB or Noc proteins. (**A**) Schematic of ParS or NBS labeling by ParB or Noc proteins. P1 locus (8XParSc or 8XNBSc) was labeled by TtParB-HaloTag or SaNoc-HaloTag and C3 repeat was visualized by dCas9-GFP/sgRNA-C3. (**B**) Sequence comparison between ParSc and NBSc. The different nucleotides between ParSc and NBSc were marked in red. (**C**) Labeling specificity of ParS or NBS labeling by ParB or Noc proteins. The 8XParSc or 8XNBSc integration site (P1 locus, red) was visualized by TtParB-HaloTag or SaNoc-HaloTag along with CRISPR-based labeling of C3 repeat (green). The scale bars are 5 μm for the cells and 1 μm for the zoom images. (**D**) The percentage of specific ParSc or NBSc labeling cells by TtParB or SaNoc. The percentage of cells with P1 foci labeled by *Tt*ParB was shown in green, and *Sa*Noc in brown. *n* = 30 cells for each group.

To examine whether Noc/NBS and ParB/ParS could be used for multicolor DNA imaging, we transfected *Sa*Noc-HaloTag into U2OS-8XParSc cells or *Tt*ParB-HaloTag into U2OS-8XNBSc cells along with dCas9-GFP/sgRNA-C3 for labeling C3 repeat (Figure 4A). The sequence comparison of ParSc and NBSc is shown in Figure 4B. There are 4 nucleotide differences between the 16 bp palindromic ParSc and NBSc sequences. As shown in Figure 4C and D, no P1 foci were detected in U2OS-8XParSc cells transfected by *Sa*Noc-HaloTag if compared to 98.1% of cells with foci positive when transfected with *Tt*ParB-HaloTag. In parallel, no P1 foci were detected in U2OS-8XNBSc cells transfected by *Tt*ParB-HaloTag if compared to 87.6% of cells with P1 foci positive when transfected with *Sa*Noc. As shown in Supplementary Figure S5, no P1 foci were detected when we transfected *Sa*Noc into U2OS-8X*Pa*ParS cells or *Pa*ParB into U2OS-8XNBSc cells. These results suggest high recognition specificity of ParB and ParS or Noc and NBS. This makes it possible to generate ParB/ParS and Noc/NBS-based multicolor DNA imaging systems, which we named mParSpot (multicolor ParSpot).

To confirm the labeling efficiency of mParSpot, we also compared the DNA labeling of the LacI/8XLacO and *Tt*ParB/8X*Tt*ParS. The 8XLacO or 8X*Tt*ParS was integrated at the P1 locus in the *PPP1R2* gene (Supplementary Figure S6A). As statistical analysis shown in Supplementary Figure S6B and S6C, only 20% of the P1 locus was specifically labeled in U2OS-8XLacO cells when transfected with LacI-HaloTag with a signal-to-noise ratio of 2.7. However, 80% of the P1 locus was specifically labeled in U2OS-8X*Tt*ParS cells when transfected with *Tt*ParB-HaloTag with a signal-to-noise ratio of 8.8. These results indicate 8X*Tt*ParS/*Tt*ParB is superior to 8XLacO/LacI in both the labeling efficiency and signal-to-noise ratio.

It has been reported that the ParB/ParS system has minimal transcription disruption (21,28). Here we compared the effect of 8XLacO, 8XParSc or U2OS-8XNBS integration (P1 locus) on the transcription repression of the *PPP1R2* gene (Supplementary Figure S7A). As shown in Supplementary Figure S7B, the mRNA level of *PPP1R2* was repressed to 27.8% in LacI-transfected U2OS-8XLacO cells. On the contrary, no repression of *PPP1R2* was observed in *Tt*ParB-transfected U2OS-8XParSc cells or *Sa*Noc-transfected U2OS-8XNBSc cells.

### Imaging pairs of genomic loci by the mParSpot system

**Figure 5.**
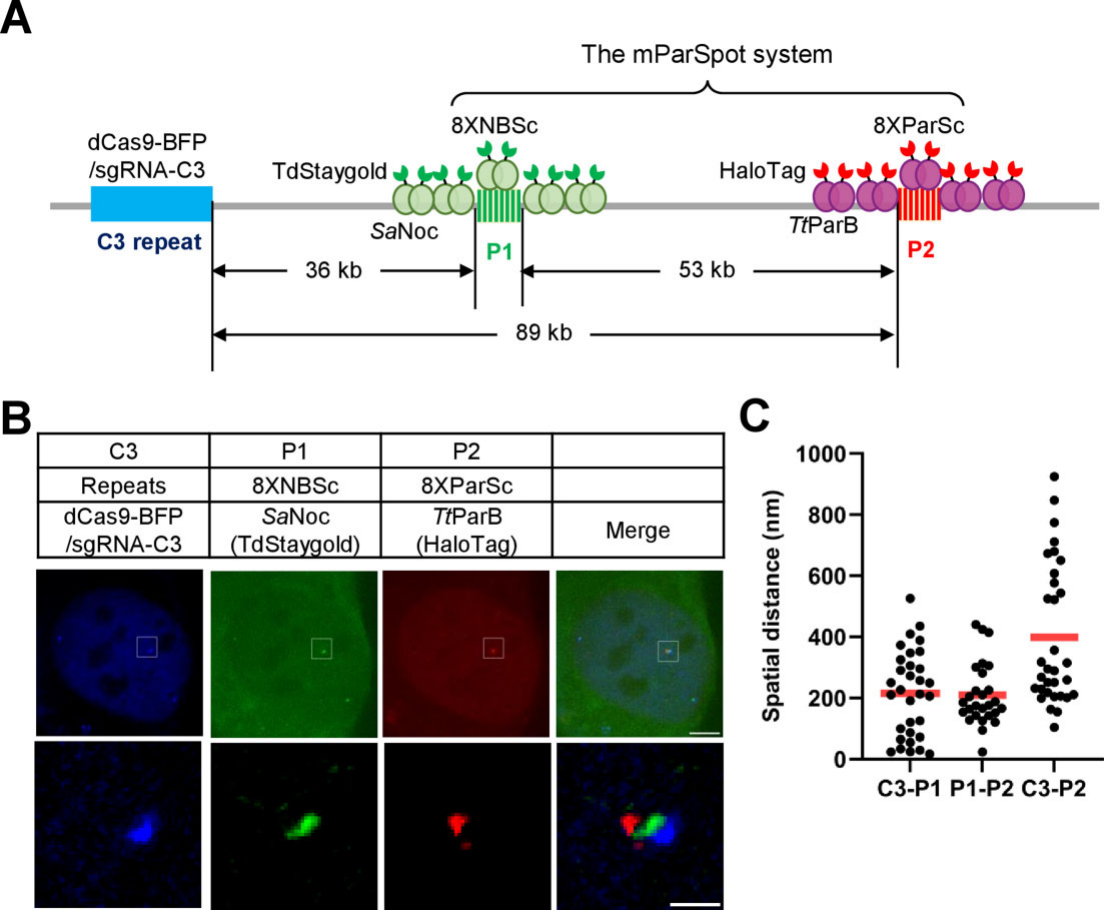
Dual color labeling of genomic loci pairs by the mParSpot system. (**A**) Schematic of dual color DNA labeling by mParSpot, the combinatory ParB–ParS and Noc-NBS system. 8XNBSc (green) or 8XParSc (purple) were inserted into 36 kb or 89 kb downstream of the C3 repeat in U2OS cells. *Sa*Noc-TdStaygold or *Tt*ParB-HaloTag was used to label the P1 locus (8XNBSc) and P2 locus (8XParSc) respectively. dCas9-GFP/sgRNA-C3 was used to visualize C3 repeat. (**B**) Dual color labeling of genomic loci by ParB–ParS and Noc-NBS. The 8XNBSc (P1 locus, green) or 8XParSc (P2 locus, red) was visualized by *Sa*Noc-TdStaygold or *Tt*ParB-HaloTag along with CRISPR-based labeling of C3 repeat (blue). The scale bars are 5 μm for the cells and 1 μm for the zoom images. (**C**) Spatial distance between genomic loci pairs. The spatial distance between C3 and P1, P1 and P2, C3 and P2 was measured. The red bar represents the average spatial distance in each group. *n* = 32 for C3-P1, 32 for P1-P2, 27 for C3-P2.

To confirm that the mParSpot could simultaneously label two target sites on a single chromosome, we knocked in 8XNBSc at the P1 locus 36 kb downstream of the C3 repeat and 8XParSc at the P2 locus 89 kb downstream of the C3 repeat into U2OS cells (Figure 5A). We transfected *Sa*Noc-TdStaygold (Staygold is a photostable and bright GFP variant (36)) to visualize the P1 locus and *Tt*ParB-HaloTag to visualize the P2 locus, along with dCas9-BFP/sgRNA-C3 for labeling C3 repeat. As shown in Figure 5B, C and Supplementary Video S2, the P1 locus (8XNBSc in green) and P2 locus (8XParSc in red) adjacent to the C3 locus (C3 repeat in blue) were visualized simultaneously in the U2OS-8XNBSc-8XParSc cells. The genomic distance between C3 and P1, P1 and P2, or C3 and P2 is 36 kb, 53 kb or 89 kb respectively. The spatial distances of these loci were measured to range from 17.3 to 526.4 nm with a mean of 215.1 nm for C3 and P1, 23.4 to 440.5 nm with a mean of 209.3 nm for P1 and P2, 104.0 to 924.2 nm with a mean of 398.9 nm for C3 and P2. Intriguingly, the mean spatial distance of P1 and P2 (209.3 nm) is similar to C3 and P1 (215.1 nm), although the genomic distance of P1 and P2 (53 kb) is 1.5-fold of C3 and P1 (36 kb). These results suggested that the spatial distances of loci pairs could deviate from genomic distances when genome organizations differ locally.

### Imaging promoter and terminator of the *MSI2* gene by the mParSpot system

**Figure 6.**
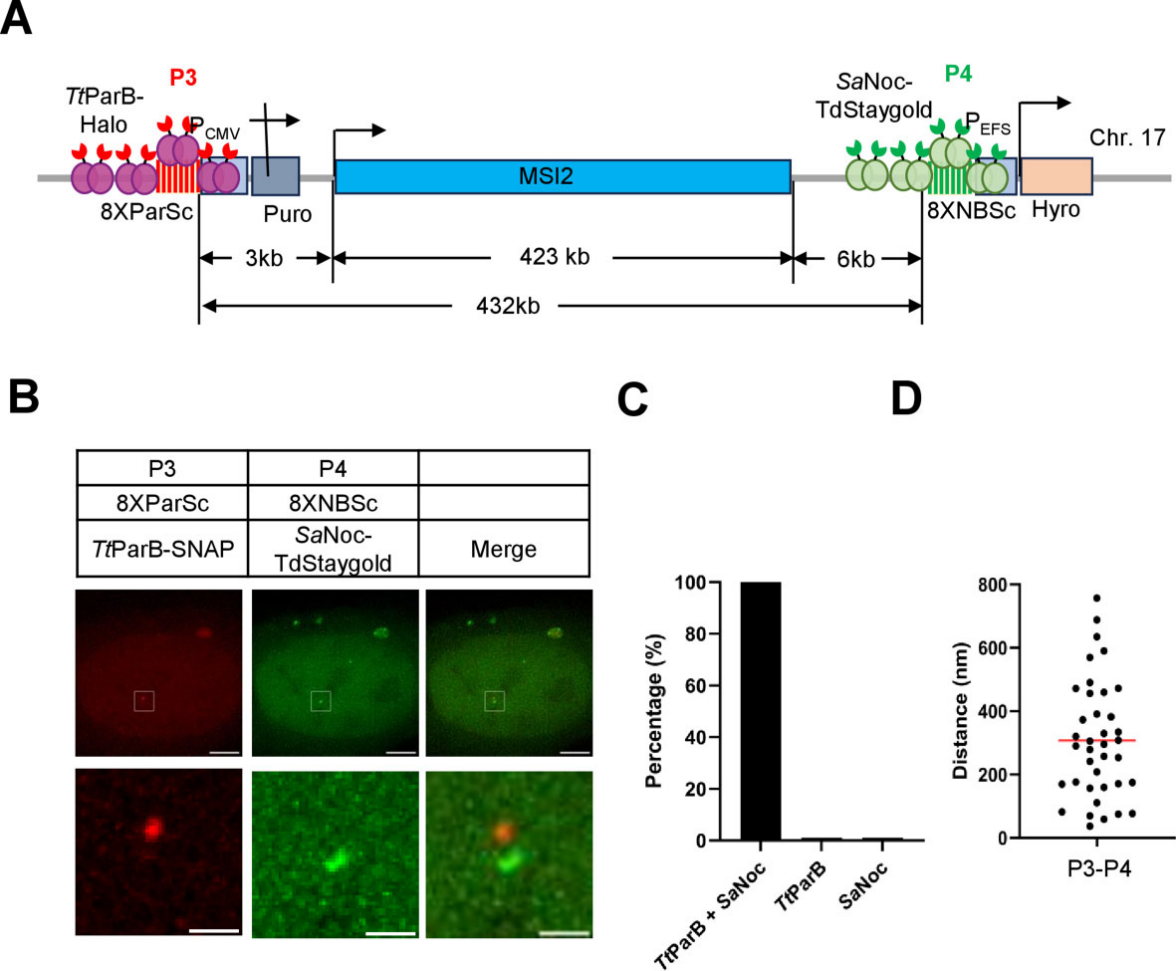
Labeling of promoter and terminator of the *MSI2* gene by mParSpot. (**A**) Schematic figure of labeling promoter and terminator of the *MSI2* gene by mParSpot. 8XParSc (P3 locus) was integrated upstream and 8XNBSc (P4 locus) was downstream of the *MSI2* gene. The genomic distances are shown below. The *MSI2* gene was located on chromosome 17. (**B**) Representative images of the *MSI2*’s promoter and terminator labeling by mParSpot. Scale bars, 5 μm for the whole cell and 1 μm for the zoom images. (**C**) The labeling efficiency of promoter and terminator of the *MSI2* gene by mParSpot. *n* = 25 cells. (**D**) The spatial distance of promoter and terminator of the *MSI2* gene. Each dot represents one cell.

To verify that mParSpot can label different genes, we inserted 8XParSc 3 kilobases upstream (P3 locus as marked the promoter region) and 8XNBSc 6 kilobases downstream (P4 locus as marked the terminator region) of the *MSI2* gene spanning 423 kb on human chromosome 17 (Figure 6A). We transfected *Tt*ParB-HaloTag to visualize the P3 locus and *Sa*Noc-TdStaygold to visualize the P4 locus. As shown in Figure 6B, the P3 locus (8XParSc) and P4 locus (8XNBSc) were visualized simultaneously in the U2OS-8XParSc-MSI2-8XNBSc cells. As statistical analysis shown in Figure 6C, almost 100% of *Tt*ParB-HaloTag and *Sa*Noc-TdStaygold transfected U2OS-8XParSc-MSI2-8XNBSc cells contain both P3 and P4 foci. The spatial distance between P3 (promoter region) and P4 (terminator region) was measured ranging from 37 to 757 nm with 307 nm on average (Figure 6D).

### Tracking promoter and terminator of the *MUC4* gene along with Pol II clusters

**Figure 7.**
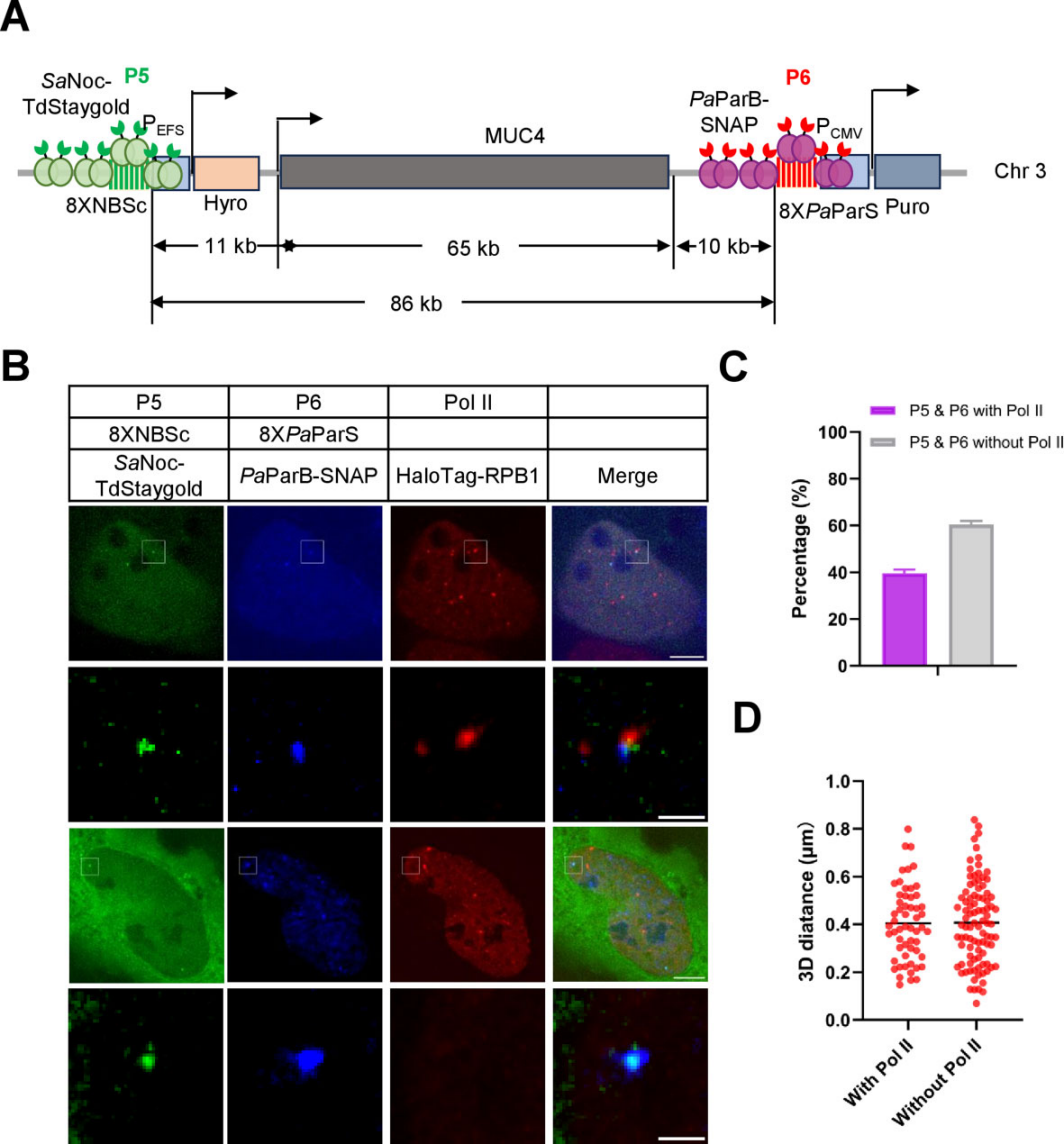
mParSpot labeling the promoter and terminator of *MUC4* along with Pol II clusters. (**A**) Schematic of mParSpot labeling the promoter and terminator of *MUC4* along with Pol II. 8XNBSc with a hygromycin expression cassette (8XNBSc-P_EFS_-Hygro) or 8X*Pa*ParS with a puromycin expression cassette (8X*Pa*ParS-P_CMV_-Puro) was inserted into 11 kb upstream or 10 kb downstream of the *MUC4* gene in U2OS cells. *Sa*Noc-TdStaygold or *Pa*ParB-SNAP was used to label the P5 locus (8XNBSc) or P6 locus (8X*Pa*ParS) along with endogenous HaloTag tagged Pol II. (**B**) mParSpot labeling of the promoter and terminator of *MUC4* along with Pol II. The 8XNBSc (P5 locus, green) or 8X*Pa*ParS (P6 locus, blue) was visualized by *Sa*Noc-TdStaygold or *Pa*ParB-SNAP along with endogenous HaloTag tagged Pol II (red). The scale bars are 5 μm for the cells and 1 μm for the zoom images. (**C**) The percentage of cells with P5, P6 and Pol II association. The percentage of cells with P5, P6 and Pol II association was shown in purple, and without P5, P6 and Pol II association in grey. *n* = 37 cells. (**D**) Comparison of spatial distances between genomic loci pairs with and without Pol II association. The spatial distance between P5 and P6 with or without Pol II association was measured. The red bar represents the average spatial distance in each group. *n* = 58 for the distance of P5 or P6 with Pol II association, 95 for the distance of P5 and P6 without Pol II association.

Genome organization in 3D is essential for transcriptional regulation and cellular function. It has been proposed that RNA Polymerase II tends to be clustered and mediates the formation of transcriptional condensates, which are associated with a set of gene promoters, enhancers or terminators for efficient RNA transcription (37,38). To examine whether we can utilize the mParSpot system for tracking the promoter and terminator of genes along with Pol II, we chose the *MUC4* gene and integration of 8XParSc at the promoter region and 8XNBSc at the terminator region. We integrated 8XNBSc 11 kb upstream (P5 locus as marked the promoter region) and 8X*Pa*ParS 10 kb downstream (P6 locus as marked the terminator region) of the *MUC4* gene and generated U2OS-8XNBSc-MUC4-8X*Pa*ParS cell lines (Figure 7A). As shown in Supplementary Figure S8, the promoter and terminator region of MUC4 are relatively stable during the 4-min tracking.

To track the association of *MUC4’*s promoter or terminator with Pol II, we knocked in HaloTag at the N-terminal of endogenous Pol II subunit RPB1, and generated U2OS-8XNBSc-MUC4-8X*Pa*ParS-Pol II-HaloTag cell lines. As shown in Figure 7B, P5 (8XNBSc) and P6 (8X*Pa*ParS) are localized adjacently regardless of their association with Pol II (red). There are 39.6% of cells showing colocalization of P5, P6, and Pol II clusters (Figure 7C). The spatial distance between P5 and P6 is ranging 70 nm to 838 nm with an average of 400 nm regardless of their colocalization with Pol II clusters (Figure 7D). We tracked the movement of P5, P6 and Pol II clusters in an eighty-second duration (Supplementary Figure S9 and Supplementary Video S3). We found that the association between P6 and Pol II clusters was more stable than the association between P5 and Pol II clusters.

## Discussion

Genome organization in space and time is essential for transcriptional regulation and cell fate determination (39). Genome organization in 3D space has been extensively studied by spatial genomics technologies such as fluorescent *in situ* hybridization (FISH) or *in situ* sequencing (ISS) (40,41). Nevertheless, genome organization in 4D (the ‘time’ is the fourth dimension) is way behind, mainly due to the lack of a robust approach for imaging genomic DNAs and their interactions over time. The fluorescence repressor operator system (FROS) is still the main approach to studying chromatin dynamics or genomic interactions in living cells. Compared to the FROS, the ParB/ParS system for DNA imaging has several advantages: (i) efficient genomic integration due to its small size; (ii) less invasive in terms of DNA damage or DNA replication; (iii) minimal transcription disruption. However, a multicolor ParB/ParS system for studying genomic interaction in mammalian cells is lacking. Here we developed the multicolor mParSpot system, which is derived from ParB/ParS and its paralogous Noc/NBS systems, allowing us to track the movement and interactions of genomic DNA pairs.

It has been reported that DNA-binding specificity for ParS and NBS is conserved within ParB and Noc Family (34). ChIP-seq data confirmed that ParB orthologs recognized the consensus ParS and Noc orthologs recognized the consensus NBS and a set of conserved residues within ParB or Noc family dictates their specificity (34). Here, we examined several ParB/ParSs and Noc/NBSs for multicolor DNA imaging in living cells. We found the combination of *Tt*ParB/ParSc and *Sa*Noc/NBSc showed high efficiency and specificity for dual-color imaging of genomic pairs.

We initially tried to develop multicolor DNA imaging by orthogonal ParB/ParS systems. Unfortunately, the low specificity of ParS recognition by orthogonal ParBs we examined prevents us from generating the multicolor DNA imaging system. Three nucleotide differences between *Pa*ParS and *Tt*ParS (Figure 1B) are not sufficient to be distinguished by their cognate ParBs. Although there are four to ten nucleotide differences between *Bc*ParS1 or *Bc*ParS2 and the other ParSs, *Bc*ParB2/*Bc*ParS2 (ANCHOR2) formed many non-specific foci regardless of the presence or absence of ParS in mammalian cells, which is excluded us from being utilized in the multicolor DNA labeling system. *Bc*ParB1/*Bc*ParS1 (ANCHOR1) was recently used in the mESCs (28), but its robustness of labeling needs to be further validated. ANCHOR3 has been used successfully in several cases (21,27,28). Unfortunately, the ANCHOR3 system is not publicly available. We believe the addition of *Pa*ParB/*Pa*ParS, *Tt*ParB/ParSc or *Sa*Noc/NBSc here will also benefit the researchers that are already using the ANCHOR3. In sum, the mParSpot system provides us with an opportunity to track the dynamics of loci pairs for a single gene or among multiple genes under physiological or pathological contexts.

## Supplementary Material

gkae134_Supplemental_Files

## Data Availability

The data underlying this article are available in the article and in its online supplementary material.

## References

[1] Rowley M.J., Corces V.G. Organizational principles of 3D genome architecture. Nat. Rev. Genet. 2018; 19:789–800. 30367165 10.1038/s41576-018-0060-8 PMC6312108

[2] Dekker J., Belmont A.S., Guttman M., Leshyk V.O., Lis J.T., Lomvardas S., Mirny L.A., O'Shea C.C., Park P.J., Ren B. et al. The 4D nucleome project. Nature. 2017; 549:219–226. 28905911 10.1038/nature23884 PMC5617335

[3] Dekker J., Alber F., Aufmkolk S., Beliveau B.J., Bruneau B.G., Belmont A.S., Bintu L., Boettiger A., Calandrelli R., Disteche C.M. et al. Spatial and temporal organization of the genome: current state and future aims of the 4D nucleome project. Mol. Cell. 2023; 83:2624–2640. 37419111 10.1016/j.molcel.2023.06.018 PMC10528254

[4] Schoenfelder S., Fraser P. Long-range enhancer-promoter contacts in gene expression control. Nat. Rev. Genet. 2019; 20:437–455. 31086298 10.1038/s41576-019-0128-0

[5] Eick D., Geyer M. The RNA polymerase II carboxy-terminal domain (CTD) code. Chem. Rev. 2013; 113:8456–8490. 23952966 10.1021/cr400071f

[6] Robinett C.C., Straight A., Li G., Willhelm C., Sudlow G., Murray A., Belmont A.S. In vivo localization of DNA sequences and visualization of large-scale chromatin organization using lac operator/repressor recognition. J. Cell Biol. 1996; 135:1685–1700. 8991083 10.1083/jcb.135.6.1685 PMC2133976

[7] Masui O., Bonnet I., Le Baccon P., Brito I., Pollex T., Murphy N., Hupé P., Barillot E., Belmont A.S., Heard E. Live-cell chromosome dynamics and outcome of X chromosome pairing events during ES cell differentiation. Cell. 2011; 145:447–458. 21529716 10.1016/j.cell.2011.03.032 PMC3756906

[8] Jacome A., Fernandez-Capetillo O. Lac operator repeats generate a traceable fragile site in mammalian cells. EMBO Rep. 2011; 12:1032–1038. 21836640 10.1038/embor.2011.158 PMC3185343

[9] Alexander J.M., Guan J., Li B., Maliskova L., Song M., Shen Y., Huang B., Lomvardas S., Weiner O.D. Live-cell imaging reveals enhancer-dependent Sox2 transcription in the absence of enhancer proximity. eLife. 2019; 8:e41769. 31124784 10.7554/eLife.41769 PMC6534382

[10] Khanna N., Zhang Y., Lucas J.S., Dudko O.K., Murre C. Chromosome dynamics near the sol-gel phase transition dictate the timing of remote genomic interactions. Nat. Commun. 2019; 10:2771. 31235807 10.1038/s41467-019-10628-9 PMC6591236

[11] Delker R.K., Munce R.H., Hu M., Mann R.S. Fluorescent labeling of genomic loci in Drosophila imaginal discs with heterologous DNA-binding proteins. Cell Rep. Methods. 2022; 2:100175. 35475221 10.1016/j.crmeth.2022.100175 PMC9017127

[12] Chen B., Gilbert L.A., Cimini B.A., Schnitzbauer J., Zhang W., Li G.W., Park J., Blackburn E.H., Weissman J.S., Qi L.S. et al. Dynamic imaging of genomic loci in living human cells by an optimized CRISPR/Cas system. Cell. 2013; 155:1479–1491. 24360272 10.1016/j.cell.2013.12.001 PMC3918502

[13] Ma H., Tu L.C., Naseri A., Huisman M., Zhang S., Grunwald D., Pederson T. Multiplexed labeling of genomic loci with dCas9 and engineered sgRNAs using CRISPRainbow. Nat. Biotechnol. 2016; 34:528–530. 27088723 10.1038/nbt.3526 PMC4864854

[14] Ma H., Tu L.C., Naseri A., Chung Y.C., Grunwald D., Zhang S., Pederson T. CRISPR-Sirius: RNA scaffolds for signal amplification in genome imaging. Nat. Methods. 2018; 15:928–931. 30377374 10.1038/s41592-018-0174-0 PMC6252086

[15] Gu B., Swigut T., Spencley A., Bauer M.R., Chung M., Meyer T., Wysocka J. Transcription-coupled changes in nuclear mobility of mammalian cis-regulatory elements. Science. 2018; 359:1050–1055. 29371426 10.1126/science.aao3136 PMC6590518

[16] Clow P.A., Du M., Jillette N., Taghbalout A., Zhu J.J., Cheng A.W. CRISPR-mediated multiplexed live cell imaging of nonrepetitive genomic loci with one guide RNA per locus. Nat. Commun. 2022; 13:1871. 35387989 10.1038/s41467-022-29343-z PMC8987088

[17] Lyu X.Y., Deng Y., Huang X.Y., Li Z.Z., Fang G.Q., Yang D., Wang F.L., Kang W., Shen E.Z., Song C.Q. CRISPR FISHer enables high-sensitivity imaging of nonrepetitive DNA in living cells through phase separation-mediated signal amplification. Cell Res. 2022; 32:969–981. 36104507 10.1038/s41422-022-00712-z PMC9652286

[18] Maass P.G., Barutcu A.R., Shechner D.M., Weiner C.L., Melé M., Rinn J.L. Spatiotemporal allele organization by allele-specific CRISPR live-cell imaging (SNP-CLING). Nat. Struct. Mol. Biol. 2018; 25:176–184. 29343869 10.1038/s41594-017-0015-3 PMC5805655

[19] Zong D., Oberdoerffer P., Batista P.J., Nussenzweig A. RNA: a double-edged sword in genome maintenance. Nat. Rev. Genet. 2020; 21:651–670. 32764716 10.1038/s41576-020-0263-7

[20] Xu H., Wang J., Liang Y., Fu Y., Li S., Huang J., Xu H., Zou W., Chen B. TriTag: an integrative tool to correlate chromatin dynamics and gene expression in living cells. Nucleic Acids Res. 2020; 48:13013–13014. 33219689 10.1093/nar/gkaa1170 PMC7736816

[21] Germier T., Kocanova S., Walther N., Bancaud A., Shaban H.A., Sellou H., Politi A.Z., Ellenberg J., Gallardo F., Bystricky K. Real-time imaging of a single gene reveals transcription-initiated local confinement. Biophys. J. 2017; 113:1383–1394. 28978433 10.1016/j.bpj.2017.08.014 PMC5627313

[22] Germier T., Audibert S., Kocanova S., Lane D., Bystricky K. Real-time imaging of specific genomic loci in eukaryotic cells using the ANCHOR DNA labelling system. Methods. 2018; 142:16–23. 29660486 10.1016/j.ymeth.2018.04.008

[23] Saad H., Gallardo F., Dalvai M., Tanguy-le-Gac N., Lane D., Bystricky K. DNA dynamics during early double-strand break processing revealed by non-intrusive imaging of living cells. PLoS Genet. 2014; 10:e1004187. 24625580 10.1371/journal.pgen.1004187 PMC3952824

[24] Komatsu T., Quentin-Froignant C., Carlon-Andres I., Lagadec F., Rayne F., Ragues J., Kehlenbach R.H., Zhang W., Ehrhardt A., Bystricky K. et al. In vivo labelling of adenovirus DNA identifies chromatin anchoring and biphasic genome replication. J. Virol. 2018; 92:e00795-18. 29997215 10.1128/JVI.00795-18 PMC6146703

[25] Chen H., Levo M., Barinov L., Fujioka M., Jaynes J.B., Gregor T. Dynamic interplay between enhancer-promoter topology and gene activity. Nat. Genet. 2018; 50:1296–1303. 30038397 10.1038/s41588-018-0175-z PMC6119122

[26] Brückner D.B., Chen H., Barinov L., Zoller B., Gregor T Stochastic motion and transcriptional dynamics of pairs of distal DNA loci on a compacted chromosome. Science. 2023; 380:1357–1362. 37384691 10.1126/science.adf5568 PMC10439308

[27] Gabriele M., Brandão H.B., Grosse-Holz S., Jha A., Dailey G.M., Cattoglio C., Hsieh T.S., Mirny L., Zechner C., Hansen A.S. Dynamics of CTCF- and cohesin-mediated chromatin looping revealed by live-cell imaging. Science. 2022; 376:496–501. 35420890 10.1126/science.abn6583 PMC9069445

[28] Platania A., Erb C., Barbieri M., Molcrette B., Grandgirard E., de Kort M.A., Meaburn K., Taylor T., Shchuka V.M., Kocanova S. et al. Competition between transcription and loop extrusion modulates promoter and enhancer dynamics. 2023; bioRxiv doi:26 April 2023, preprint: not peer reviewed 10.1101/2023.04.25.538222. PMC11641109 39671497

[29] Jalal A.S.B., Le T.B.K. Bacterial chromosome segregation by the ParABS system. Open Biology. 2020; 10:200097. 32543349 10.1098/rsob.200097 PMC7333895

[30] Lin D.C., Grossman A.D. Identification and characterization of a bacterial chromosome partitioning site. Cell. 1998; 92:675–685. 9506522 10.1016/s0092-8674(00)81135-6

[31] Bartosik A.A., Lasocki K., Mierzejewska J., Thomas C.M., Jagura-Burdzy G. ParB of Pseudomonas aeruginosa: interactions with its partner ParA and its target parS and specific effects on bacterial growth. J. Bacteriol. 2004; 186:6983–6998. 15466051 10.1128/JB.186.20.6983-6998.2004 PMC522188

[32] Li H., Angelov A., Pham V.T., Leis B., Liebl W. Characterization of chromosomal and megaplasmid partitioning loci in Thermus thermophilus HB27. BMC Genomics. 2015; 16:317. 25909452 10.1186/s12864-015-1523-3 PMC4409726

[33] Adams D.W., Wu L.J., Errington J. Nucleoid occlusion protein Noc recruits DNA to the bacterial cell membrane. EMBO J. 2015; 34:491–501. 25568309 10.15252/embj.201490177 PMC4331003

[34] Jalal A.S.B., Tran N.T., Stevenson C.E., Chan E.W., Lo R., Tan X., Noy A., Lawson D.M., Le T.B.K. Diversification of DNA-binding specificity by permissive and specificity-switching mutations in the ParB/Noc protein family. Cell Rep. 2020; 32:107928. 32698006 10.1016/j.celrep.2020.107928 PMC7383237

[35] Jalal A.S.B., Tran N.T., Wu L.J., Ramakrishnan K., Rejzek M., Gobbato G., Stevenson C.E.M., Lawson D.M., Errington J., Le T.B.K. CTP regulates membrane-binding activity of the nucleoid occlusion protein Noc. Mol. Cell. 2021; 81:3623–3636. 34270916 10.1016/j.molcel.2021.06.025 PMC8429893

[36] Hirano M., Ando R., Shimozono S., Sugiyama M., Takeda N., Kurokawa H., Deguchi R., Endo K., Haga K., Takai-Todaka R. et al. A highly photostable and bright green fluorescent protein. Nat. Biotechnol. 2022; 40:1132–1142. 35468954 10.1038/s41587-022-01278-2 PMC9287174

[37] Zheng M., Tian S.Z., Capurso D., Kim M., Maurya R., Lee B., Piecuch E., Gong L., Zhu J.J., Li Z. et al. Multiplex chromatin interactions with single-molecule precision. Nature. 2019; 566:558–562. 30778195 10.1038/s41586-019-0949-1 PMC7001875

[38] Cramer P. Organization and regulation of gene transcription. Nature. 2019; 573:45–54. 31462772 10.1038/s41586-019-1517-4

[39] Zheng H., Xie W. The role of 3D genome organization in development and cell differentiation. Nat. Rev. Mol. Cell Biol. 2019; 20:535–550. 31197269 10.1038/s41580-019-0132-4

[40] Bouwman B.A.M., Crosetto N., Bienko M. The era of 3D and spatial genomics. Trends Genet. 2022; 38:1062–1075. 35680466 10.1016/j.tig.2022.05.010

[41] Bintu B., Mateo L.J., Su J.H., Sinnott-Armstrong N.A., Parker M., Kinrot S., Yamaya K., Boettiger A.N., Zhuang X. Super-resolution chromatin tracing reveals domains and cooperative interactions in single cells. Science. 2018; 362:eaau1783. 30361340 10.1126/science.aau1783 PMC6535145

