# Supplementary material for: Tracking pairwise genomic loci by the ParB–ParS and Noc-NBS systems in living cells
Source: Nucleic Acids Res. 2024 Feb 27;52(9):4922–34. doi: 10.1093/nar/gkae134 (PMC11109969; doi:10.1093/nar/gkae134)
Supplement: gkae134_Supplemental_Files [file gkae134_supplemental_files.zip › mParSpot-NAR revision-SI-Final.pdf]

Tracking Pairwise Genomic Loci by the ParB-ParS and Noc-NBS Systems in Living Cells

Xiaohui He[1], Yuxi Tan[1], Ying Feng[1], Yadong Sun[2], Hanhui Ma[1*]

[1]Gene Editing Center, School of Life Science and Technology, ShanghaiTech University, Shanghai, China.

[2]School of Life Science and Technology, ShanghaiTech University, Shanghai, China.

*Correspondence should be addressed to Hanhui Ma: mahh@shanghaitech.edu.cn

Supplementary information:

Supplementary Figures

Supplementary Tables

Supplementary Videos

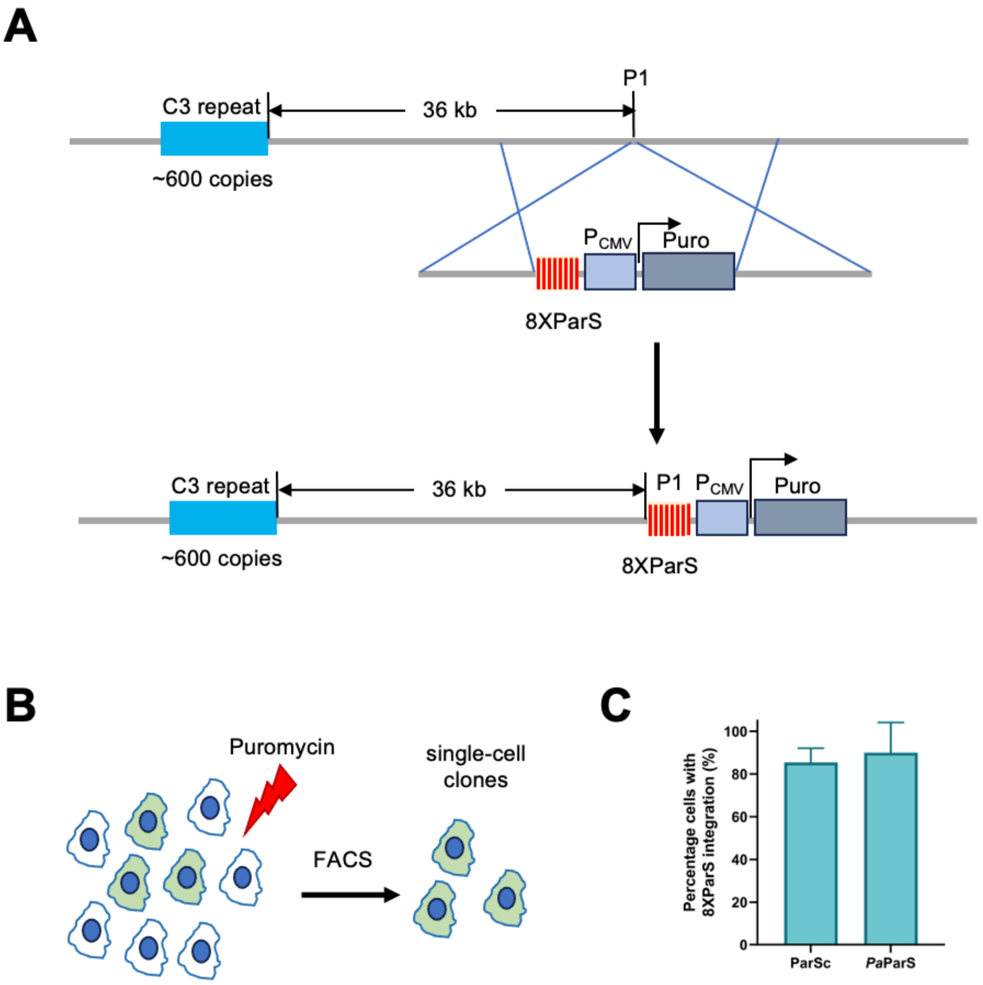

Figure S1. Integration of 8XParS adjacent to C3 repeat in the U2OS cells.

A) Schematic of integration of 8XParS adjacent to C3 repeat. ParS octet along with a puromycin expression cassette (8XParS-P_{CMV}-Puro) was inserted into the P1 locus, which is 36 kilobases downstream of the C3 repeat, by the Cas9-mediated homologous recombination in U2OS cells.

B) Clonal selection by FACS. Donor plasmids containing 8XParS-P_{CMV}-Puro along with Cas9/sgRNA-P1 were transfected into U2OS cells. Puromycin was used to kill these cells without transfected and FACS was applied for the single-cell selection. Clonal cells were used to validate the efficiency of integration.

C) Integration efficiency of 8XParS into U2OS cells. The integration efficiency was estimated by counting the positive clones with 8XParS from single-cell selection.

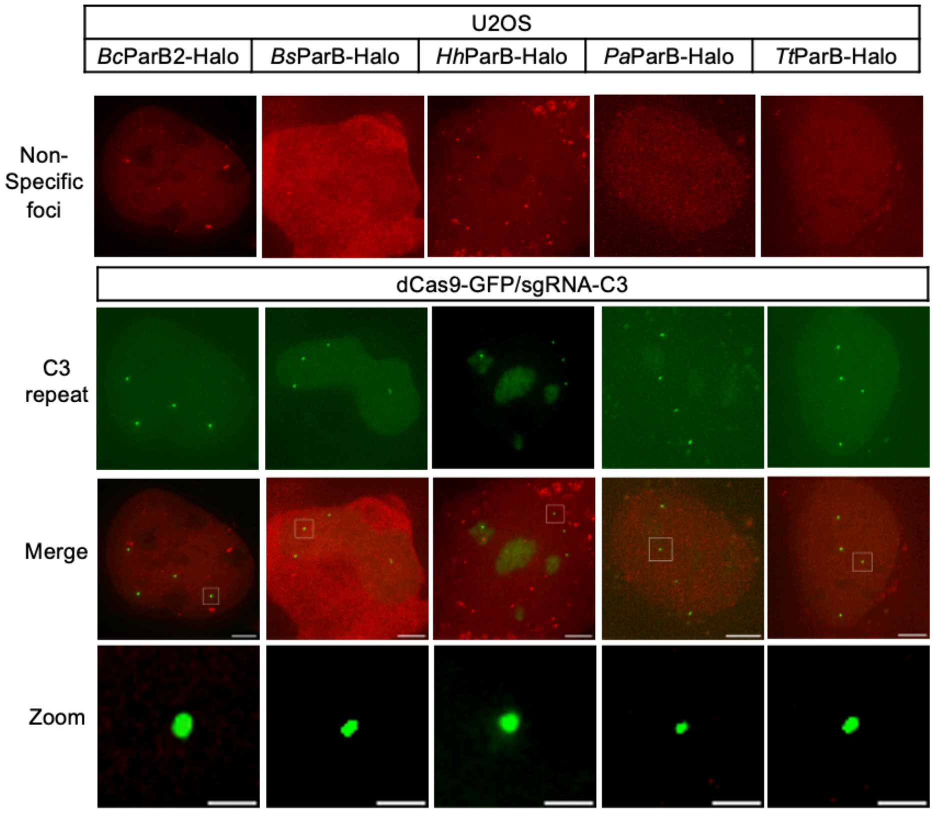

Figure S2. Non-specific foci formation of orthogonal ParBs in U2OS cells.

Orthogonal ParB-HaloTag (red) along with dCas9-GFP/sgRNA-C3 (green) was transfected into U2OS cells. The non-specific foci in these transfected cells were examined. The scale bars are 5 μm for the cells and 1 μm for the zoom images.

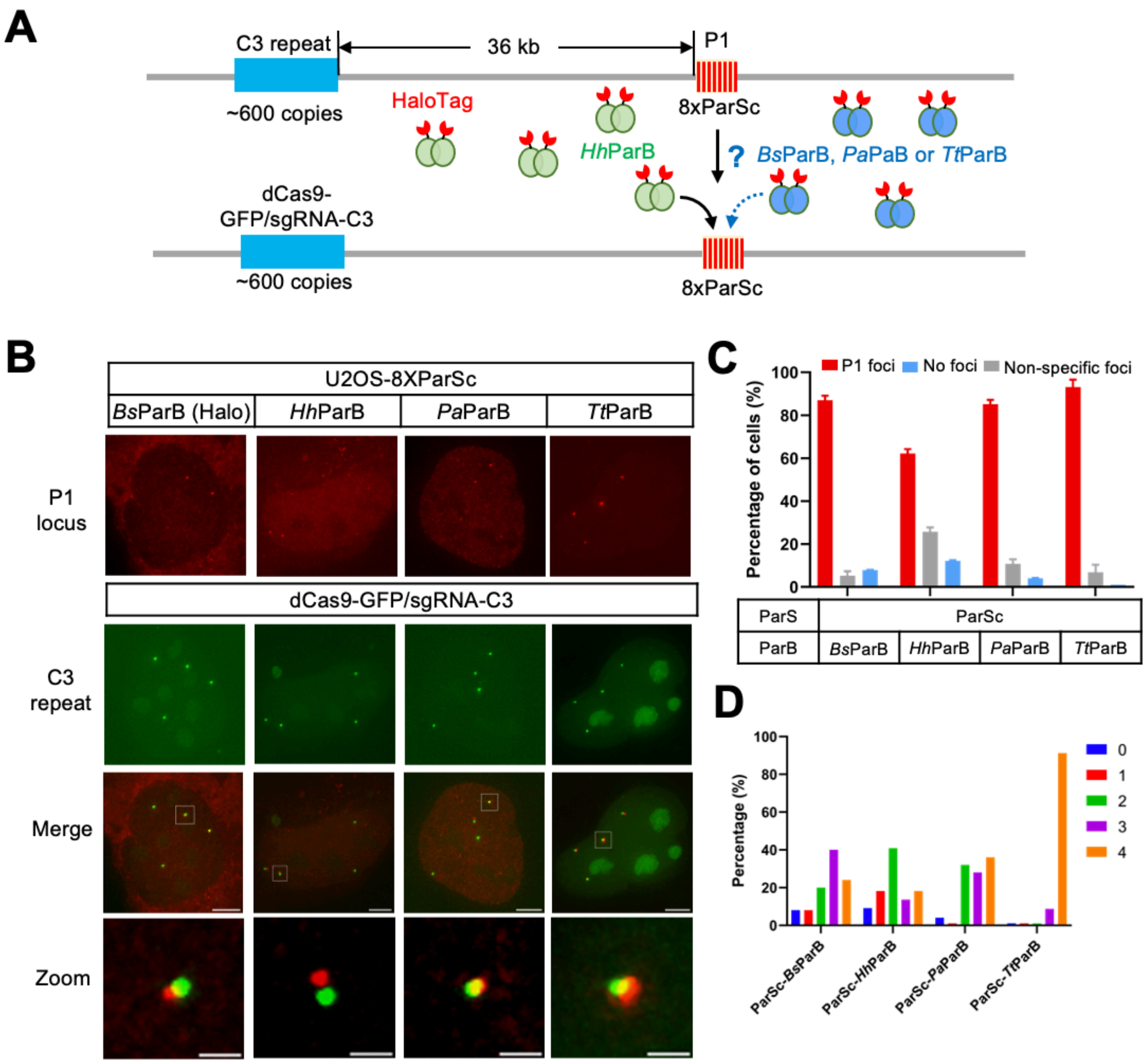

Figure S3. The specificity of ParSc recognition by orthogonal ParBs.

A) Schematic of ParSc labeling by the orthogonal ParBs. 8XParSc was inserted into 36 kilobases downstream of the C3 repeat in U2OS cells. P1 locus (8XParSc) was labeled by orthogonal ParB-HaloTag and C3 repeat was visualized by dCas9-GFP/sgRNA-C3.

B) Labeling specificity of ParSc by orthogonal ParBs. The 8XParSc integration site (P1 locus, red) was visualized by orthogonal ParB-HaloTag along with CRISPR-based labeling of C3 repeat (green). Scale bars: 5 μm for the cells and 1 μm for zoom images.

C) The percentage of specific labeling cells by ParSc and orthogonal ParBs. The percentage of cells with specific P1 foci was shown in red, no foci in blue, and non-specific foci in grey. n=25 cells in each group.

D) The detection efficiency of ParSc by *Bs*ParB, *Hh*ParB, *Pa*ParB and *Tt*ParB. n = 25 cells for *Bs*ParB, 22 for *Hh*ParB, 25 for *Pa*ParB, and 26 cells for *Tt*ParB.

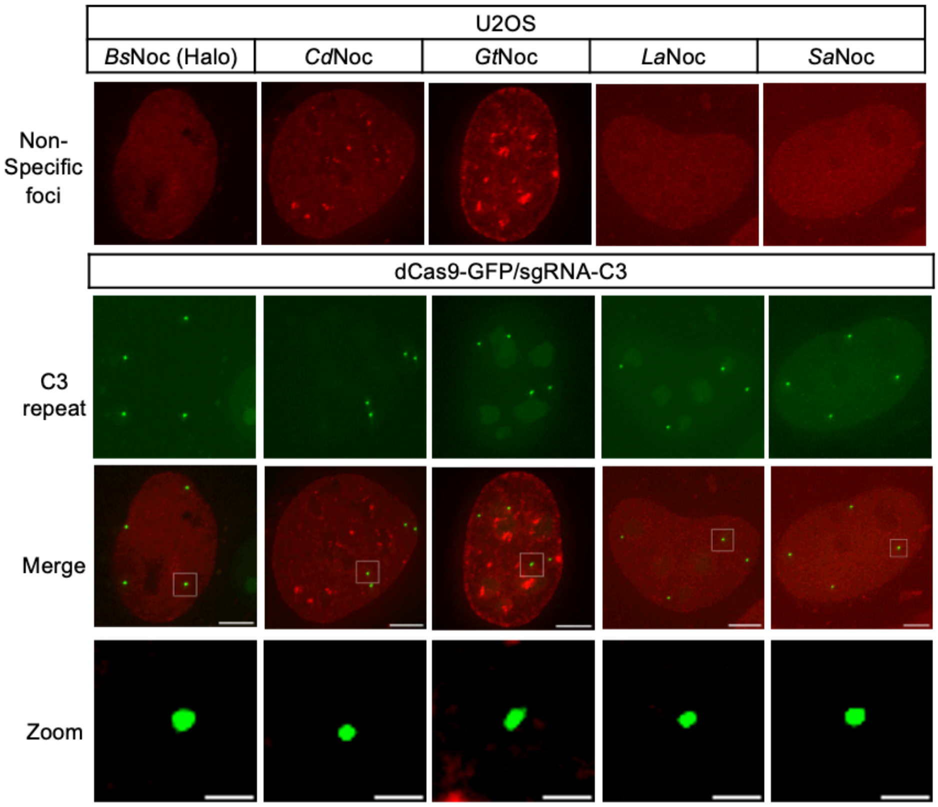

Figure S4. Non-specific foci formation of orthogonal Noc in U2OS cells.

Orthogonal Noc-HaloTag (red) along with dCas9-GFP/sgRNA-C3 (green) was transfected into U2OS cells. The non-specific foci in these transfected cells were examined. The scale bars are 5 μm for the cells and 1 μm for the zoom images.

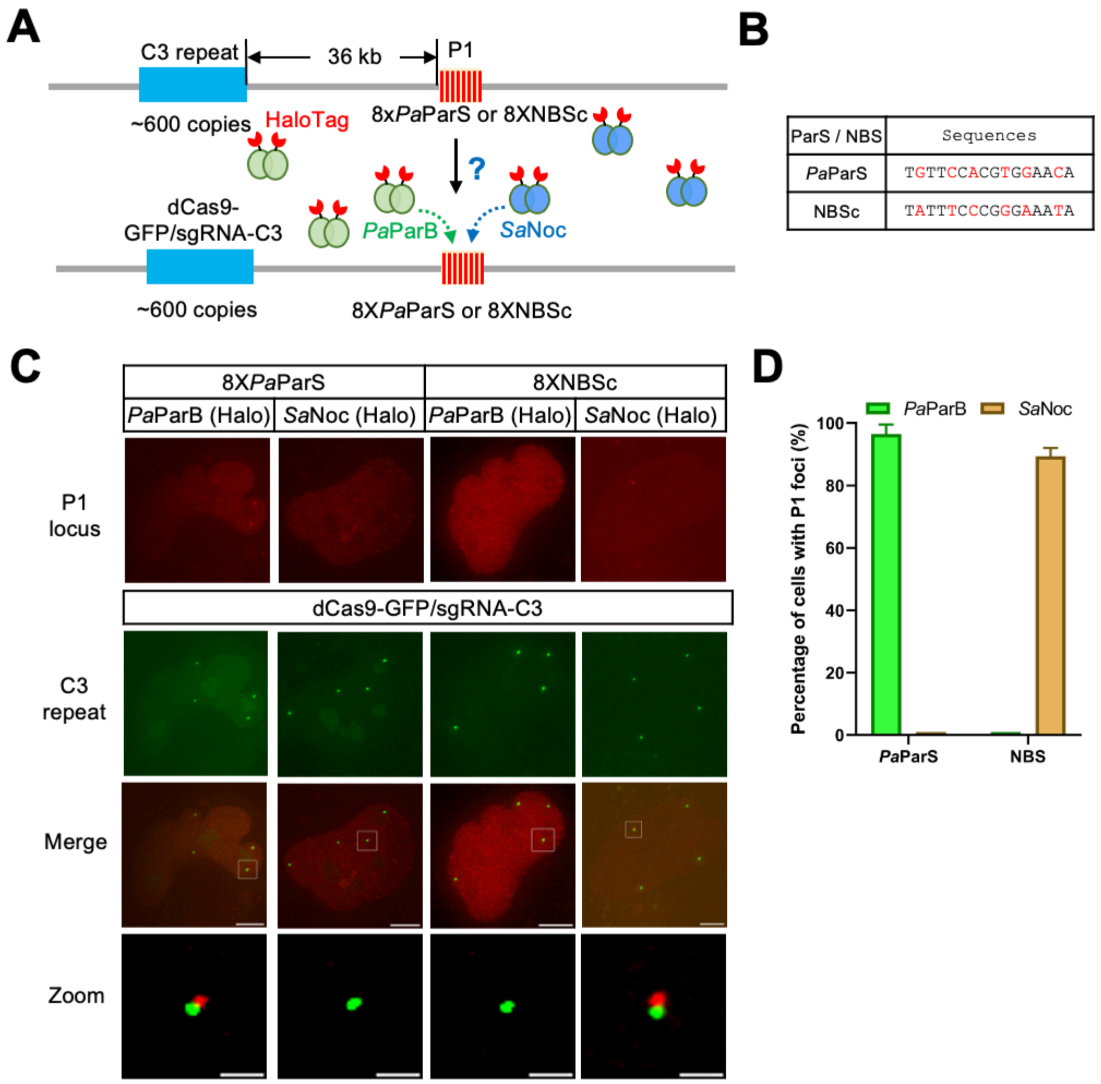

Figure S5. The specificity of *Pa*ParS or NBSc recognition by *Pa*ParB or *Sa*Noc.

A) Schematic of *Pa*ParS or NBSc labeling by *Pa*ParB or *Sa*Noc proteins. P1 locus (8X*Pa*ParS or 8XNBSc) was labeled by *Pa*ParB-HaloTag or *Sa*Noc-HaloTag and C3 repeat was visualized by dCas9-GFP/sgRNA-C3.

B) Sequence comparison between *Pa*ParS and NBSc. The different nucleotides between PaParS and NBSc were marked in red.

C) Labeling specificity of *Pa*ParS or NBSc labeling by *Pa*ParB or *Sa*Noc. The 8X*Pa*ParS or 8XNBSc integration site (P1 locus, red) was visualized by *Pa*ParB-HaloTag or *Sa*Noc-HaloTag along with CRISPR-based labeling of C3 repeat (green). The scale bars are 5 µm for the cells and 1 µm for the zoom images.

D) The percentage of specific *Pa*ParS or NBSc labeling cells by *Pa*ParB or *Sa*Noc. The percentage of cells with P1 foci labeled by *Pa*ParB was shown in green, and *Sa*Noc in brown. n=20 cells in each group.

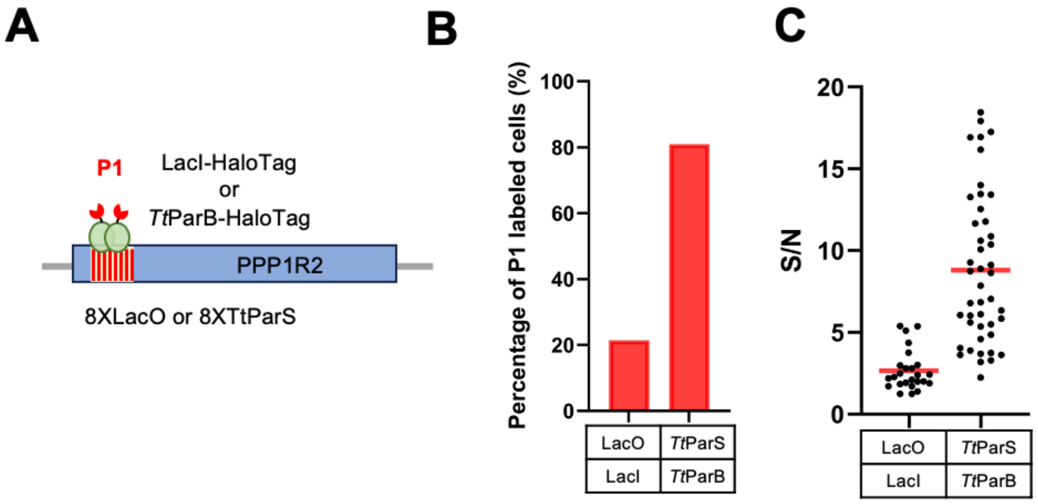

Figure S6. The comparison of DNA labeling by LacO/LacI and *Tt*ParS/*Tt*ParB.

A) Schematic diagram of comparison method. 8XLacO or 8X*Tt*ParS was integrated at P1 locus in PPP1R2 gene. LacI-HaloTag or *Tt*ParB-HaloTag was used to label 8XLacO or 8X*Tt*ParS.

B) The percentage of P1 locus labeled cells by LacO/LacI or *Tt*ParS/*Tt*ParB. n =28 cells for LacO/LacI, 21 cells for *Tt*ParS/*Tt*ParB.

C) The signal to noise ratio of LacO/LacI and *Tt*ParS/*Tt*ParB. n =25 for LacO/LacI, 45 for *Tt*ParS/*Tt*ParB. The red line indicates the mean value.

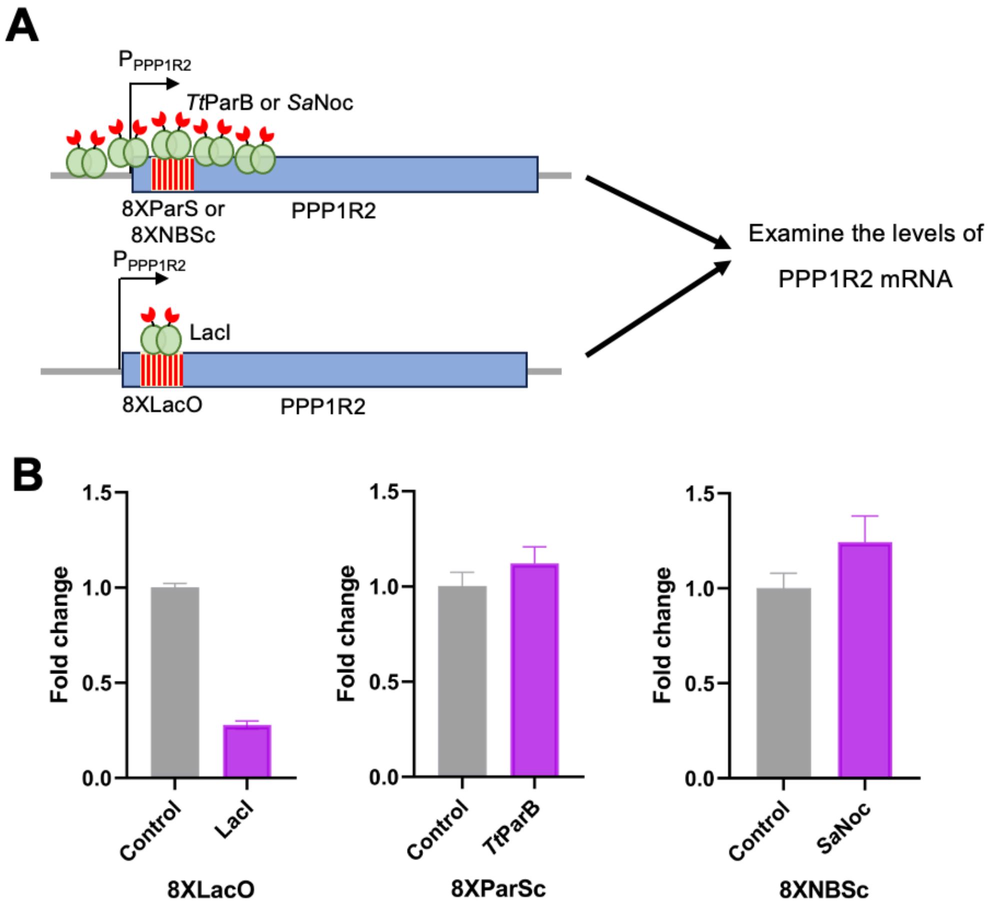

Figure S7. The influence of mParSpot on gene transcription.

A) The diagram of detecting the interference of mParSpot and LacI/LacO on gene expression. 8XParS, 8XNBSc or 8XLacO was integrated into PPP1R2 gene. *Tt*ParB, *Sa*Noc or LacI was transfected to see its impact on the level of PPP1R2 mRNA.

B) Fold change of PPP1R2 mRNA level in 8XLacO/LacI group, 8XParSc/*Tt*ParB group, and 8XNBSc/*Sa*Noc group.

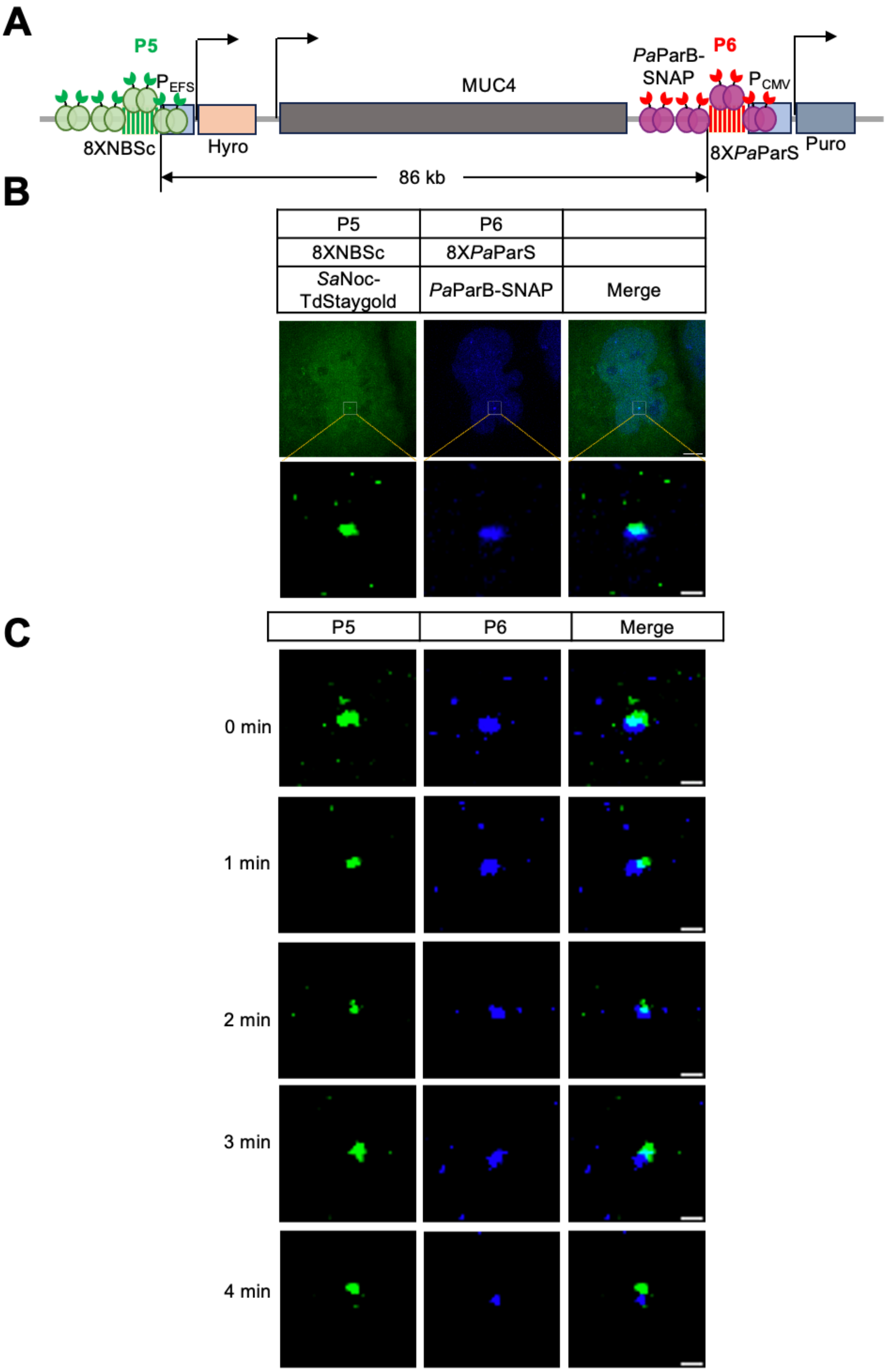

Figure S8. Time-lapse of promoter and terminator of the *MUC4* gene by mParSpot.

A) The diagram of labeling strategy.

B) Representative images of P5 and P6 labeled by *Sa*Noc-tdStaygold (green) and *Pa*ParB-

SNAP (blue) respectively. Scale bars, 5 μm for the cells and 0.5 μm for the zoom images.

C) The dynamics of P5 (green) and P6 (blue) over 4 minutes. Scale bars, 0.5 μm.

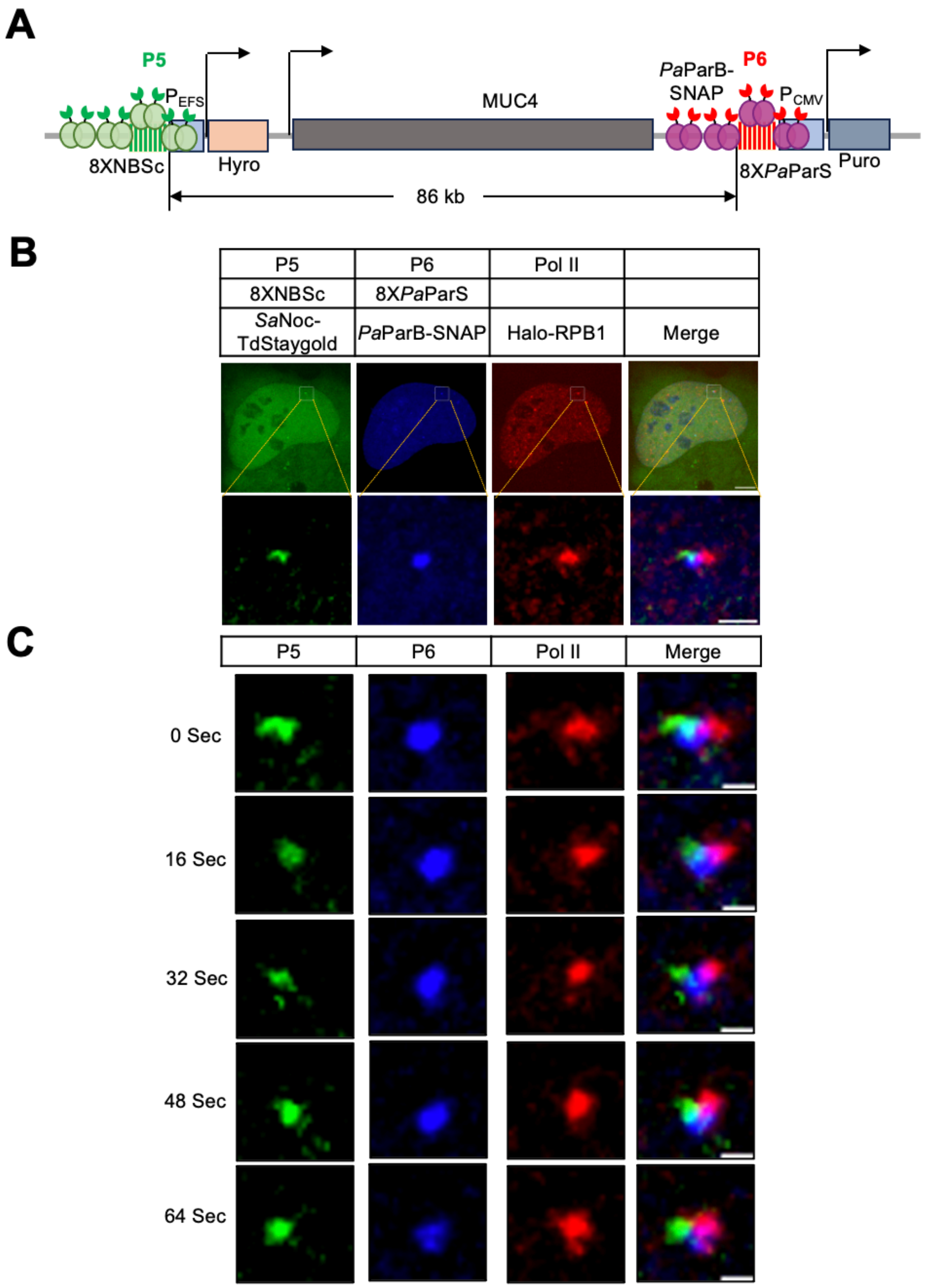

Figure S9. mParSpot Tracking of promoter and terminator along with Pol II.

A) Schematic of mParSpot labeling *MUC4*'s promoter and terminator along with Pol II. 8XNBSc with a hygromycin expression cassette (8XNBSc-P_{EFS}-Hygro) or 8XPaParS with a puromycin expression cassette (8X*Pa*ParS-P_{CMV}-Puro) was inserted upstream or downstream of the MUC4 gene spanning 86 kilobases. *Sa*Noc-TdStaygold or *Pa*ParB-SNAP was used to label the P5 locus (8XNBSc) or P6 locus (8X*Pa*ParS) along with

endogenous HaloTag tagged Pol II.

B) Colocalization of P5, P6 and Pol II condensates. The 8XNBSc (P5 locus, green) or 8X*Pa*ParS (P6 locus, blue) was visualized by *Sa*Noc-TdStaygold or *Tt*ParB-HaloTag along with endogenous HaloTag tagged Pol II (red). The scale bars are 5 μm for the cells and 1 μm for the zoom images.

C) Tracking the dynamics of P5, P6 and Pol II condensates. Three color time-lapse images of P5, P6 and Pol II were captured for 80 seconds. The images at 0, 16, 32, 48, and 64 second time points were shown. The scale bars are 0.5 μm.

Video S1. Track P1 locus by *Tt*PatB.

The video is shown in a total time of 100 sec. Images were cropped to 50 X 50 pixels. Scale bar, 1 μm. The playback rate is 15 frames per second. Red: P1 locus and Green: C3 repeat.

Video S2. Tracking the dynamics of P1 and P2 loci pair by the mParSpot system.

The video is shown in a total time of 50 sec. Images were cropped to 50 X 50 pixels. Scale bar, 0.5 μm. The playback rate is 15 frames per second. Green: P1 locus, Red: P2 locus, and Purple: C3 repeat.

Video S3. mParSpot Tracking dynamics of promoter and terminator along with Pol II.

The video is shown in a total time of 80 sec. Images were cropped to 50 X 50 pixels. Scale bar, 1 μm. The playback rate is 15 frames per second. Green: P5 locus, Blue: P6 locus, and Red: Pol II cluster.

Table S1. The sequences of 8XParS and 8XNBSc.

The 8XParS consists of ParS sequences (capital letters) and linker sequences. The 8XNBSc consists of NBSc sequence (capital letters) and linker sequences.

8X*Bc*ParS1	cgcgaccgacgtgtgcgTTTATGCGCATAAAcgcactcagcttgacatggcgctaaatatcggcgTTTATGCGCATAAAcaccgtgtggaatacacccggcatcgcgtcctgTTTATGCGCATAAAgccatccagattgtactcaccgatggacacacgTTTATGCGCATAAAcccttggcagggtcaacagcaccttgggattgcgtctcgactcgcggccgccgcgaccgacgtgtgcgTTTATGCGCATAAAcgcactcagcttgacatggcgctaaatatcggcgTTTATGCGCATAAAcaccgtgtggaatacacccggcatcgcgtcctgTTTATGCGCATAAAgccatccagattgtactcaccgatggacacacgTTTATGCGCATAAAcccttggcagggtcaacagcaccttgggattg
8X*Bc*ParS2	cgcgaccgacgtgtgcGTTGTCACGTGACAACcccggcatcgcgtcctGTTGTCACGTGACAACcagcaccttgggattGTTTGTCACGTGACAACaatcgatatccggcgaCTTGTCACGTGACAACcggctcttcctcgttagaattccctcgaatactgagttGTTGTCACGTGACAACtggcgctaaatatcggGTTGTCACGTGACAACtcaccgatggacacaCTTTGTCACGTGACAACtcatcttcccaggtagCTTGTCACGTGACAACggactagccctaaagcaaatg
8X*Bs*ParS	cgcgaccgacgtgtgcTGTTACACGTGAAACAcgcactcagcttgacatggcgctaaatatcggcGTTTCACGTGAAACAcaccgtgtggaatacacccggcatcgcgtcctTGTTACACGTGAAACAgccatccagattgtactcaccgatggacacacTGTTACACGTGAAACAcccttggcagggtcaacagcaccttgggattggaattcgcggccgccgcgaccgacgtgtgcTGTTACACGTGAAACAcgcactcagcttgacatggcgctaaatatcggcGTTTCACGTGAAACAcaccgtgtggaatacacccggcatcgcgtcctTGTTACACGTGAAACAgccatccagattgtactcaccgatggacacacTGTTACACGTGAAACAcccttggcagggtcaacagcaccttgggattg
8X*Pa*ParS	cgcgaccgacgtgtgcTGTTCCACGTGGAACCcgcactcagcttgacatggcgctaaatatcggTGTTCCACGTGGAACAcaccgtgtggaatacacccggcatcgcgtcctTGTTCCACGTGGAACAgccatccagattgtactcaccgatggacacacTGTTCCACGTGGAACCccttggcagggtcaagaattccgcgaccgacgtgtgcTGTTCCACGTGGAACCcgcactcagcttgacatggcgctaaatatcggTGTTCCACGTGGAACAcaccgtgtggaatacacccggcatcgcgtcctTGTTCCACGTGGAACAgccatccagattgtactcaccgatggacacacTGTTCCACGTGGAACCcccttggcagggtcaa
8X*Tt*ParS	cgcgaccgacgtgtgcTGTTTCACGGGAAACAcgcactcagcttgacatggcgctaaatatcggcTGTTTCACGGGAAACAcaccgtgtggaatacacccggcatcgcgtcctTGTTTCACGGGAAACAgccatccagattgtactcaccgatggacacacTGTTTCACGGGAAACAcccttggcagggtcaacagcaccttgggattgcgtctcgactcgcggccgccgcgaccgacgtgtgcTGTTTCACGGGAAACAcgcactcagcttgacatggcgctaaatatcggcTGTTTCACGGGAAACAcaccgtgtggaatacacccggcatcgcgtcctTGTTTCACGGGAAACAgccatccagattgtactcaccgatggacacacTGTTTCACGGGAAACAcccttggcagggtcaacagcaccttgggattg
8XParSc	cgcgaccgacgtgtgcTGTTTCACGTGAAACAcgcactcagcttgacatggcgctaaatatcggcTGTTTCACGTGAAACAcaccgtgtggaatacacccggcatcgcgtcctTGTTTCACGTGAAACAgccatccagattgtactcaccgatggacacacTGTTTCACGTGAAACAcccttggcagggtcaacagcaccttgggattgcgtctcgactcgcggccgcTGTTTCACGTGAAACAcgcactcagcttgacatggcgctaaatatcggcTGTTTCACGTGAAACAcaccgtgtggaatacacccggcatcgcgtcctTGTTTCACGTGAAACAgccatccagattgtactcaccgatggacacacTGTTTCACGTGAAACAcccttggcagggtcaacagcaccttgggattg
8XNBSc	cgcgaccgacgtgtgcgaTATTTCCCGGGAAATAtccgcactcagcttgacatggcgctaaatatcggcgaTATTTCCCGGGAAATAtccaccgtgtggaatacacccggcatcgcgtcctgaT

	ATTTCCCGGGAAATAtcgccatccagattgtactcaccgatggacacacgaTATTTCCCGGGAAATAtccccttggcagggtcaacagcaccttgggattgcgtctcgactcgcggccgccgcgaccgacgtgtgcgaTATTTCCCGGGAAATAtccgcactcagcttgacatggcgctaaatatcggcgaTATTTCCCGGGAAATAtccaccgtgtggaatacacccggcatcgcgtcctgaTATTTCCCGGGAAATAtcgccatccagattgtactcaccgatggacacacgaTATTTCCCGGGAAATAtccccttggcagggtcaacagcaccttgggattg

Table S2. sgRNA spacer sequence for genomic integration and C3 labeling.

SgRNAs for genomic integration	
P1-SgRNA	GCTCCTCGTCGACATTCCCG
P2-SgRNA	AGTCACTTCCGCTCACATGG
P3-SgRNA	AAGCTCCTTCCCAAATCATC
P4-SgRNA	AAGGCATTCCCGCTACACTG
P5-SgRNA	TGGCACTCCTGGTTTCGTGC
P6-SgRNA	TGAATGGAAACGAATTCCGT
Pol II-SgRNA	CCTGCCTCCGCCATGCACGG
SgRNAs for C3 labeling	
C3 repeat-SgRNA	TGATATCACAG

Table S3. Primer sequence for genotyping of stable cell lines.

Primers	
P1-F	ACTAATGGACTTCCGGTCGTT
P1-R	CTCTGCCTAGACGGGTTGTG
P2-F	GCAGGGGATTGCCAAGTAGT
P2-R	TCAGCTGGAATGTTCACTATGCCAC
P3-F	CCTGATGCAGGGGCTACATT
P3-R	TAGCCTTCCAAAGGCATCGC
P4-F	TAAACCCCAAAGGTCCTGCG
P4-R	TTTGTTGACTCAGGTAGGCACC
P5-F	CTCAAAGGTTGACCTGCTATTGACTG
P5-R	CCCGCATATAAGAAACACCAGCTC
P6-F	CGTCAACAAGAACAACACAAATTAGAAG
P6-R	GAGCCCAGTCAAAGCCGCTGAG
P1-middle-R	CCCCGTAATTGATTACTATTAATAACTAGT.
